# MR Spectroscopic Imaging of Hyperpolarized 129‐Xenon in the Dissolved‐Phase to Determine Regional Chemical Shifts of Hyperoxia in Healthy Porcine Lungs

**DOI:** 10.1002/nbm.70063

**Published:** 2025-05-18

**Authors:** Michael Vaeggemose, Mattias H. Kristensen, Mohsen Redda, Esben S. S. Hansen, Oliver Rodgers, Guilhem J. Collier, Graham Norquay, Jim M. Wild, Christoffer Laustsen, Rolf F. Schulte

**Affiliations:** ^1^ MR Research Centre, Department of Clinical Medicine Aarhus University Aarhus Denmark; ^2^ GE HealthCare Brondby Denmark; ^3^ POLARIS Group, Section of Imaging, Division of Clinical Medicine University of Sheffield Sheffield UK; ^4^ GE HealthCare Munich Germany

**Keywords:** arterial oxygen partial pressure, blood oxygenation saturation, chemical shift imaging, dissolved‐phase imaging, hyperpolarized xenon gas, magnetic resonance imaging, porcine animal model

## Abstract

Lung MRI with hyperpolarized xenon (^129^Xe) gas reveals key characteristics of pulmonary physiology such as ventilation and alveolar‐capillary gas transfer. Magnetic resonance spectroscopic imaging (MRSI) offers insights into regional oxygenation saturation (sO_2_) through chemical shift changes related to xenon–hemoglobin binding. The similarity between porcine and human anatomy and physiology, particularly in terms of lung volume, airway structure, and alveolar‐capillary microstructure, offers the opportunity to investigate physiological effects linked to oxygen supply using ^129^Xe MRSI. We hypothesize that ^129^Xe MRSI can detect regional chemical shift changes related to red blood cell oxygenation and arterial oxygen partial pressure (p_a_O_2_) in a porcine model. Imaging was performed on a 3‐T clinical MRI scanner on four healthy pigs mechanically ventilated at fractional inspired oxygen levels (FiO_2_) of 40% and 100%. Dissolved‐phase images were acquired using a 3D Cartesian MRSI sequence with a spherical sampling pattern in a matrix size of 28 × 28 × 6. A spectrally tailored RF pulse excited the dissolved and gaseous phases with flip angles of 10° and 0.1°, respectively. Repetition time was 7.4 ms resulting in a total acquisition time of 18 s. In addition, ^129^Xe ventilation, pulmonary anatomical scans, dynamic contrast‐enhanced perfusion, and arterial blood gas were measured at each FiO_2_. Pair‐wise comparisons were performed between inspired oxygen levels, along with linear regression analysis of p_a_O_2_ and dissolved‐phase chemical shift imaging. Porcine lung lobes were segmented, and two‐way ANOVA were performed to evaluate regional effects of oxygen concentrations. Arterial blood gas and cardiopulmonary measures showed an increase in p_a_O_2_ with the increase in FiO_2_. Ventilation defect percentage and perfusion metrics did not significantly change with higher oxygen concentration. Dissolved‐phase ratios of red blood cells (RBC) to membrane increased with higher oxygen concentration. Increasing inspired oxygen resulted in a lower RBC chemical shift and increased linewidth, indicating RBC measures are sensitive to p_a_O_2_. Simple linear regression analysis of RBC chemical shift and a multiple linear regression model including linewidth were applied for regional p_a_O_2_ maps. Regional effects of oxygen were confirmed in the segmented lung lobes. Dissolved‐phase ^129^Xe chemical shift of RBC decreased linearly with p_a_O_2_ in healthy porcine lungs. Regional chemical shift, linewidth, and signal ratio changes were determined in dissolved‐phase imaging of RBC at 40% and 100% FiO_2_. Our data suggest that regional p_a_O_2_ prediction is possible with a multiple linear regression model including RBC chemical shift and linewidth as combined effect of oxygen across animal lung lobes affects regions differently.

## Graphical abstract

Dissolved‐phase hyperpolarized xenon gas red blood cells (RBC) chemical shift decreases with increasing arterial oxygen partial pressure (p_a_O_2_) in healthy porcine lungs. Our data suggest a strong predictive value for estimating p_a_O_2_ based on linear regression analysis of RBC chemical shift images at 40% and 100% inspired oxygen levels (F_i_O_2_).

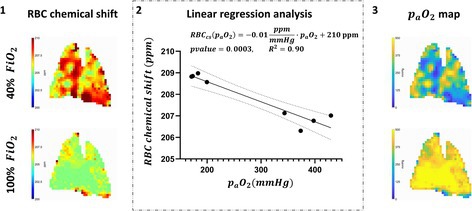

**Abbreviations**
AMARES: advanced method for accurate, robust, and efficient spectral
COPD: chronic obstructive pulmonary disease
CRLB: Cramér–Rao lower bounds
CS: chemical shift
DCE: dynamic contrast‐enhanced
eCO_2_
: exhaled CO_2_
FOM: first‐order moment
HR: heart rate
HCT: hematocrit
HPV: hypoxic pulmonary vasoconstriction
IPF: idiopathic pulmonary fibrosis
FiO_2_
: inspired oxygen level
IDEAL: iterative decomposition with echo asymmetry and least‐squares estimation
LW: linewidth
MRSI: magnetic resonance spectroscopic imaging
mPAP: mean pulmonary artery pressure
MTT: mean transit time
MLR: multiple linear regression fit
pO_2_
: oxygen partial pressure
sO_2_
: oxygenation saturation
ODC: oxygen–hemoglobin dissociation curve
pCO_2_
: partial pressure of carbon dioxide
PF: plasma flow
PEEP: positive end‐expiratory pressure
PCV‐VG: pressure‐controlled ventilation‐volume guaranteed mode
PA: alveolar pressure
Pa: pulmonary arterial oxygen pressure
p_a_O_2_
: arterial oxygen partial pressure
Pv: pulmonary venous pressure
RBC: red blood cells
SLR: simple linear regression fit
SEOP: spin exchange optical pumping
sccm: standard cubic centimeters per minute
TRICKS: time‐resolved imaging of contrast kinetics
VDP: ventilation defect percentage
VD: volume of distribution
^129^Xe: hyperpolarized xenon gas

## 1 Introduction

Hyperpolarized xenon (^129^Xe) gas MRI and magnetic resonance spectroscopic imaging (MRSI) can detect key characteristics of pulmonary physiology such as ventilation and gas transfer from the alveoli to the pulmonary capillaries [1, 2]. Inhaled hyperpolarized ^129^Xe gas dissolves in alveolar tissue (e.g., lung parenchyma) and via diffusional uptake into the red blood cells (RBCs) and blood plasma in the capillaries, giving rise to three distinct spectral resonance lines:gas phase; lung tissue and plasma (membrane); RBCs. The xenon absorption follows a similar gas transfer path of oxygen making it possible to measure multiple aspects of regional lung function during a single breath‐hold. Dissolved‐phase ^129^Xe imaging is enabled by a large gas pool with a long T_1_ (~20 s [3]) and the rapid exchange between gas and dissolved compartments. However, accurate delineation of the dissolved resonances remains technically challenging due to short T_2_* (~1 ms at 3 T [4]) and low signal intensity (1%–2% of gas phase) [5]. ^345^ Several dissolved‐phase imaging methods have been proposed for clinical use, including 3D radial 1‐point Dixon [6], multipoint 3D radial spectroscopic imaging [5, 7], 3D radial iterative decomposition with echo asymmetry and least‐squares estimation (IDEAL) [8], 2D spiral MRSI [9, 10], and 3D MRSI [11, 12].

MRSI encodes full spectra using sequential phase encoding along each spatial dimension. Although this is time consuming, short TR can recover some encoding efficiency. Although the achievable resolution is lower as compared with methods using imaging readout gradients, measuring the full spectrum improves robustness and provides additional insights into changes in chemical shifts and linewidths of the resonances in the lungs. ^129^Xe MRSI can provide valuable insight into lung physiology by detecting regional changes in the dissolved‐phase chemical shift [6]. Previous studies have shown that a reduction in blood oxygen saturation (sO_2_) decreases dissolved‐phase RBC chemical shift, as observed in healthy subjects [13, 14], and in patients with idiopathic pulmonary fibrosis (IPF) [8, 12, 15] or chronic obstructive pulmonary disease (COPD) [12].

^129^Xe dissolved‐phase imaging has been applied in several preclinical models including rodents, guinea pigs, dogs, sheep, rabbits, and pigs [16, 17, 18]. Pigs have four lobes in the right lung and two lobes in the left lung while the human lung has three lobes in the right lung and two lobes in the left lung [19]. The porcine anatomy and physiology are similar to humans in terms of lung volume, airway structure and mucosa [20, 21] with the addition of a bronchus emerging from the trachea supplying the cranial lobe of the right lung [22]. In addition, the porcine oxygen‐hemoglobin dissociation curve (ODC) is subject to minor variation as to that obtained in humans [23]. This provides the opportunity to apply a porcine animal model to regulate physiological effects of oxygen partial pressure (pO_2_) and sO_2_ more easily in validation of MRSI applications.

Previous studies focused on the RBC chemical shift decrease in humans during apnea from breath‐holds of up to 40 s and oxygenation in whole blood samples. Apnea and whole blood drop in sO_2_ was exponentially related to dissolved‐phase RBC chemical shift [13]. Xenon T_1_ is sensitive to hemoglobin (oxygen binding) changes and decreases with pO_2_ [13, 24, 25]. Previous results are related to the ODC, which is commonly used to model the relationship between arterial oxygen partial pressure (p_a_O_2_) and sO_2_. Nevertheless, apnea is an extreme condition where oxygen saturation is lowered past normal conditions. Importantly, although significant work has been done to characterize RBC chemical shift changes under hypoxic conditions, there is a lack of data evaluating the reverse effect—how increased arterial oxygen partial pressure under normal or hyperoxic conditions (sO_2_ ~100%) influences dissolved‐phase RBC chemical shifts. Furthermore, the regional effects of oxygenation on p_a_O_2_ mapping using ^129^Xe MRSI remain poorly understood, particularly in preclinical models that closely resemble human pulmonary physiology.

The aim of this study was to investigate if dissolved‐phase ^129^Xe spectroscopic imaging can be used to assess p_a_O_2_ in healthy porcine lungs ventilated at fractional inspired oxygen levels of 40% and 100%. This work represents a critical step in advancing dissolved‐phase hyperpolarized ^129^Xe imaging as a tool for assessing regional lung oxygenation, with potential implications for studying pulmonary diseases and informing clinical practices.

## 2 Methods

### 2.1 Hyperpolarized ^129^Xe Gas Preparation

For all experiments ^129^Xe gas was polarized to approximately 30% polarization levels using a spin exchange optical pumping (SEOP) polarizer (POLARIS, University of Sheffield, Sheffield, UK) [26]. The gas mixture of 3% isotopically enriched xenon (86% ^129^Xe), 10% N_2_, and 87% He flowed through a glass cell (volume = 3534 cm^3^; temperature = 130°C; total gas pressure = 2 bars) at a flow rate of 2000 standard cubic centimeters per minute (sccm). After exiting the glass cell, the hyperpolarized ^129^Xe was cryogenically separated in a liquid nitrogen‐cooled glass spiral and collected in its frozen state over a time of 9 min (xenon ~800 mL). Following xenon gas distillation, the sample was sublimated using hot water (~40°C) and dispensed into a 1‐L Tedlar bag. For ^129^Xe lung ventilation images, the 3% xenon gas mixture was polarized, collected without the use of cryogenic separation, and dispensed directly into a 1‐L Tedlar bag.

### 2.2 Animal Model and Anesthetics

The study examined four female Danish Landrace pigs weighing approximately 40 kg. The pigs were anesthetized through an ear vein catheter (fentanyl and propofol, dose 8 and 18 mL/h, respectively), intubated and put on a mechanical ventilator at fractional inspired oxygen levels (FiO_2_) of 40% and 100% with a continuous flow of 5400 mL/min in pressure‐controlled ventilation‐volume guaranteed mode (PCV‐VG). Throughout the scan sessions, vital cardiopulmonary parameters were measured, including heart rate (HR), systolic and diastolic blood pressure and mean arterial pressure (mPAP), respiration frequency, positive end‐expiratory pressure (PEEP), and tidal volume and exhaled CO_2_ (eCO_2_).

This study complied with institutional and national guidelines and was approved by the Danish Animal Inspectorate before initiation.

### 2.3 Animal Handling and Xenon Gas Administration

Animals were positioned in a supine position, intubated, and catheters were placed in the femoral vein and artery during preparation in the operating theater. Preparation enables controlled mechanical ventilation, injection of gadolinium contrast and blood gas measures throughout the scan session.

Before transport, the pigs were wrapped in plastic to insulate, fixate limbs, and to reduce the risk of equipment contamination at the MR facilities. During transportation to the MR scanner, the pigs were manually ventilated with a self‐inflating bag. Upon arrival, the tracheal tube was connected to a mechanical ventilator with FiO_2_ set to 40%. Mechanical ventilation and intravenous anesthesia were maintained through ventilation and plastic tubes. Anesthesia was monitored by HR, blood pressure, and end tidal CO_2_.

**FIGURE 1:**
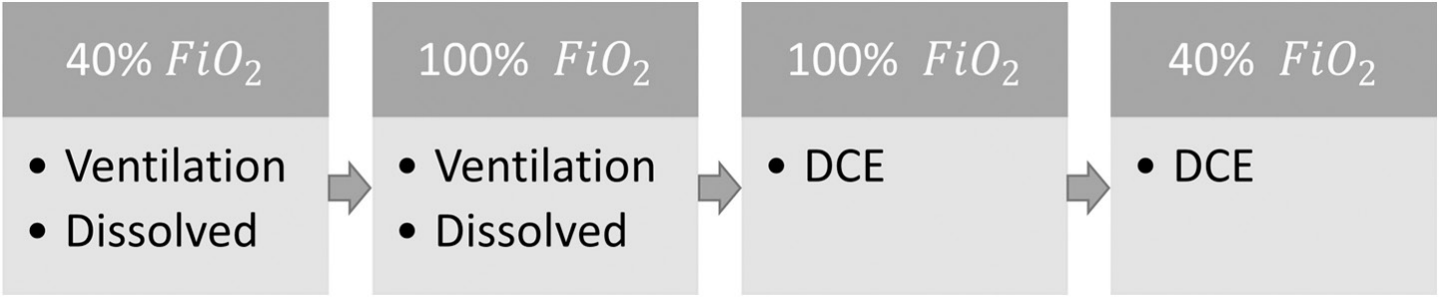
Scan session overview included a 20‐min wait between interventions to ensure oxygen saturation and pulmonary gadolinium contrast washout. Interventions are illustrated with grey boxes. DCE = dynamic contrast‐enhanced imaging, dissolved = dissolved‐phase imaging, FiO_2_ = fractional inspired oxygen level.

After transportation and between each intervention a 20‐min wait was included to ensure oxygen saturation and pulmonary gadolinium contrast washout. The interventions are illustrated with boxes in Figure 1. This resulted in a total scan time of 2 h including MRI sequences and 2‐min rests between each xenon gas sequence.

Xenon gas was administrated by unplugging the mechanical ventilation system from the tracheal tube and switching to the Tedlar bag. Functional residual capacity was achieved by waiting approximately 2 s before connecting the bag to the tracheal tube. Xenon gas was administered to the lungs in a steady flow with a gentle pressure on the bag emptying it in 3 s to avoid damaging the lungs with excessive pressure.

### 2.4 Arterial Blood Gas Measures

A clinical blood gas analyzer (Radiometer, ABL80, UK) was used to analyze arterial blood samples, drawn peripherally in the femoral artery, before acquiring the dissolved‐phase images. The blood gas analyzer calculates sO_2_ and hematocrit (HCT) from measured p_a_O_2_, partial pressure of carbon dioxide (pCO_2_), and acidity (pH).

### 2.5 Image Acquisition

Xenon images of porcine lungs were acquired on a 3‐T MRI scanner (MR750, GE HealthCare, Waukesha, WI, USA) using a ^129^Xe transmit‐receive quadrature vest coil (Clinical MR Solutions, Brookfield, WI, USA) tuned to 35.3 MHz.

A 3D coronal balanced steady‐state free precession (b‐SSFP) sequence was used to acquire ventilation images [27]; voxel size of 5 × 5 × 10 mm^3^, matrix size of 80 × 80 × 30, repetition time (TR) = 3.2 ms, echo time (TE) = minimum (~1 ms), flip angle (FA) = 10°, bandwidth = 31.25 kHz, and total acquisition time of 6 s.

**FIGURE 2:**
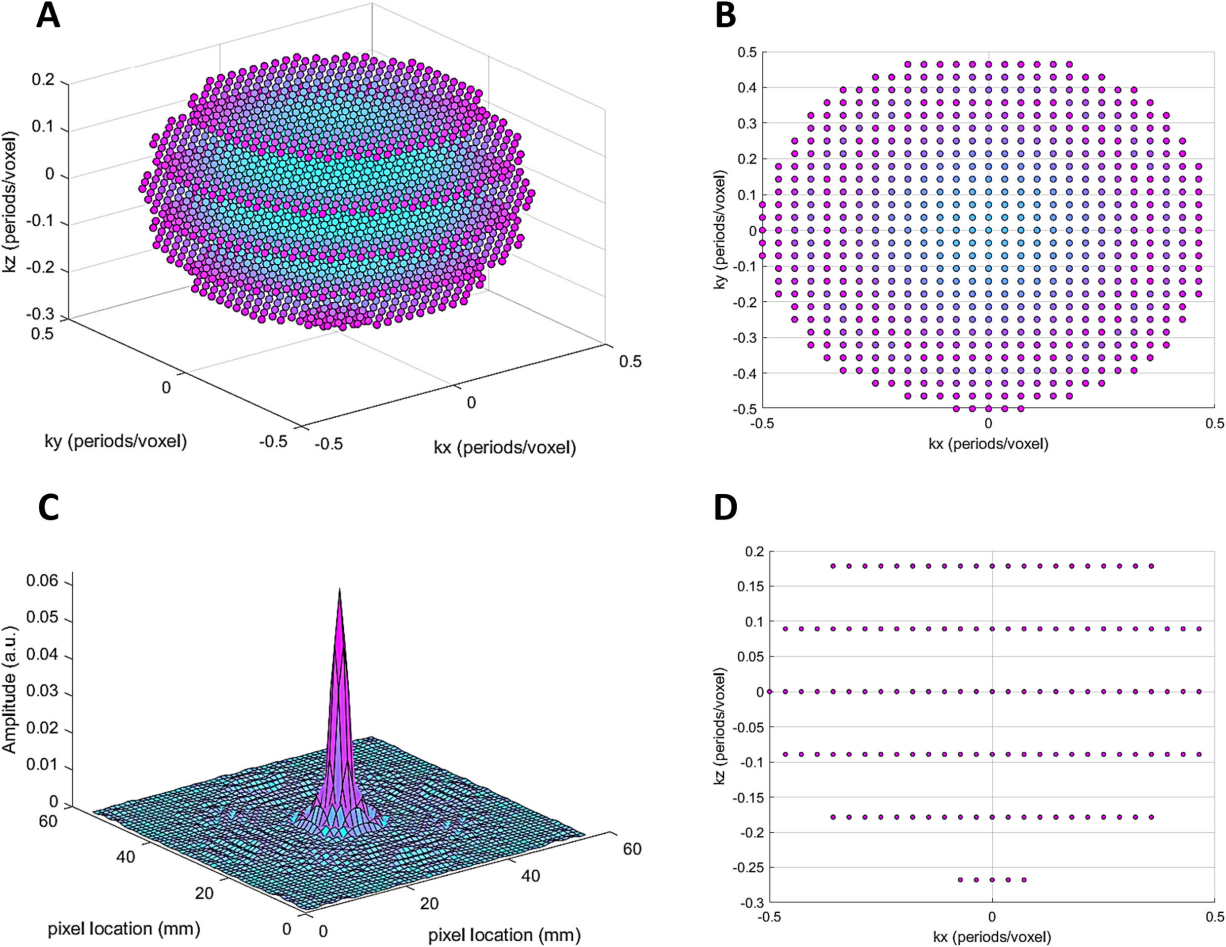
Illustration of the 3D spherical undersampled Cartesian sampling trajectory in 3D (A), through the center plane at kz = 0 periods/voxel (B), through the center plane at ky = 0 periods/voxel (D), and the point spread function (PSF) (C). k‐space normalized in all three dimensions to ± 0.5 periods/voxel.

A 3D Cartesian MRSI trajectory with a spherical sampling pattern was designed with a matrix size of 28 × 28 × 6 covering a field‐of‐view (FOV) of 40 × 40 × 20 cm^3^ with a total of 2416 excitations. A spectrally tailored RF pulse with a duration of 0.6 ms and partial self‐refocusing was designed to excite the dissolved and gas phases with FAs of 10° and 0.1° and passbands of 500 and 200 Hz, respectively [11]. The repetition time was set to 7.4 ms resulting in a total acquisition time of 18 s, suitable for a single breath‐hold in pigs. Dissolved xenon signal in the heart and large pulmonary vessels was saturated to less than 6% by applying 20 initial dummy scans with a 30° flip angle prior to data acquisition. Data were acquired with 88 samples at a bandwidth of 20 kHz and spectrally zero‐filled to 256 samples, corresponding to a spectral resolution of 78 Hz (2.21 ppm). Acquired data were spatially zero‐filled by a factor of 2. Figure 2A,B,D illustrates the Cartesian uniform spherical center‐out sampling trajectory in 3D, through the center plane in two orientations. Figure 2C illustrates the k‐space normalized point spread function.

As anatomical reference, coronal 3D T_1_‐weighted (T_1_w) proton images were acquired from the same volume as xenon ventilation using a 3D SSFP imaging sequence with FOV = 40 × 40 × 20 cm^3^, matrix = 256 × 256 × 128, FA = 3°, TR = 3.4 ms, and TE = 2.1 ms on the scanner's body coil within a breath‐hold after administration of a 1‐L Tedlar bag of ambient air corresponding to the volume of administrated xenon gas.

Lung perfusion was assessed by dynamic contrast‐enhanced (DCE) MRI, with injection of gadolinium (Dotarem, Guerbet, Villepinte, France) (0.2 mmol/kg at ∼5 mL/s intravenously) using time‐resolved imaging of contrast kinetics (TRICKS) [28]. TRICKS was acquired with a matrix size of 256x256x88, reconstructed to an image resolution of 1.5 × 1.5 × 3 mm^3^, TR = 2.216 ms, TE = 0.764 ms, and FA = 20°. The temporal resolution was 1.2 s for each phase (depending on the number of partitions), and a total of 45 phases were acquired within 1:05 min. Prior to injecting the contrast agent, a background scan was obtained (5 s), followed by a breath‐hold (25 s) during the subsequent phases. Perfusion images were acquired using a 32‐channel Body Array Coil (MR750, GE HealthCare, Waukesha, WI, USA).

### 2.6 Image Processing

Ventilation defect percentage (VDP) was calculated as the proportion of total lung cavity volume (TCV) without ventilation [29]. T_1_w images were segmented to calculate TCV using active contours cluster segmentation in ITK‐SNAP 3.8.0 [30]. The ^129^Xe images were segmented to calculate the ventilated lung volume (VV) by a 10% threshold of the maximum signal intensity. The ventilation percentage difference was calculated using Equation (1):

(1) $$\mathrm{VDP}=\left(1-\frac{\mathrm{VV}}{\mathrm{TCV}}\right)\cdot 100\%$$

**FIGURE 3:**
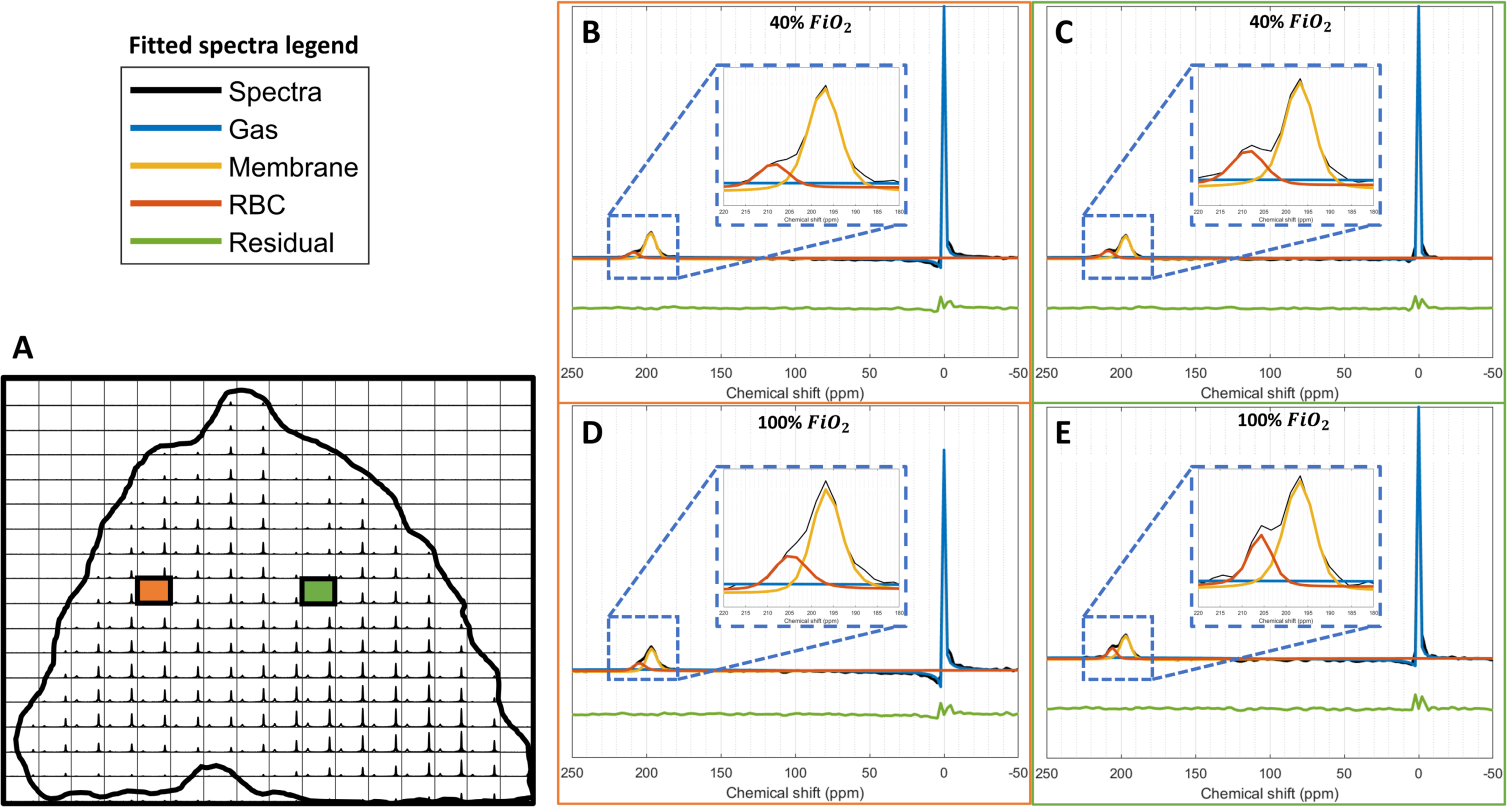
Fitted MRSI spectra at the two fractional inspired oxygen levels (FiO_2_). (A) MRSI spectra of the center slice with indication of selected spectra (B–E) close to the heart (green) and in the lung periphery (orange). Fitted spectra of FiO_2_ at 40% (B,D) and 100% (C,E) are illustrated at the right with real values of original spectra (black) and AMARES fitted spectra of the dissolved phases (membrane = orange, RBC = red) and gas (blue). Fitting residual values are shown in green. Zoom inserts at 185–215 ppm are shown to improve visualization of the dissolved‐phase region (dotted blue line). RBC = red blood cells, AMARES = advanced method for accurate, robust, and efficient spectral.

Gas, membrane, and RBC signal, phases, chemical shifts, and linewidths were determined with advanced method for accurate, robust, and efficient spectral (AMARES) fitting using OXSA [31], an open‐source magnetic resonance spectroscopy analysis toolbox in MATLAB. AMARES is a linear least‐squares fitting algorithm incorporating prior knowledge for each signal component [32]. The prior knowledge constrains the fitting algorithm by setting initial values and bounds of chemical shift, linewidth, amplitude, phase, and intrinsic relationships between the peaks (gas, membrane, and RBC). The prior knowledge also defined peak line shapes using Lorentzian (gas and RBC) and Voigt (membrane) distributions [33]. The prior knowledge file was initially optimized by solving the least‐squares of the acquired dissolved‐phase free‐induction decay (FID) signals average. The resulting amplitudes, chemical shifts, and phases are then used as the starting values for the main fitting routine. Zero and first‐order phase correction was applied to each FID automatically prior to AMARES fitting to compensate for the transmit to receive delay, receiver dead‐time, and individual peak phase difference. Cramér–Rao lower bounds (CRLB) were determined from the fitting and can be used as an uncertainty measure [34]. An example of AMARES fitted spectra from the acquired dissolved phases and gas spectra of a voxel close to the heart (green) and in the central right lung (orange) is shown in Figure 3. Signal‐to‐noise ratio (SNR) was determined from the AMARES fitted signal mean values of gas, RBC, and membrane divided separately by the standard deviation of the noise. The noise was measured at frequencies far from the three main xenon resonance frequencies (−150 to −250 ppm). Following fitting, differences between dissolved and gas phase excitation flip angles was corrected, and compensated for changes in T_2_* decay by multiplying exp (TE/T_2_*) to the corresponding signal amplitude. Quantified dissolved‐phase components are masked with a threshold of 0.6 times the gas signal mean value. Dissolved‐phase images were reconstructed and analyzed in MATLAB R2020a (MathWorks, Natick, MA, USA) using the MNS Research Pack created by GE HealthCare.

Perfusion was quantified in UMMPerfusion with a pixel‐by‐pixel deconvolution approach on the T_1_w DCE‐MRI images providing maps of mean transit time (MTT), plasma flow (PF), and volume of distribution (VD). The arterial‐input‐function was estimated using a region‐of‐interest placed in the pulmonary trunk. UMMPerfusion is an open‐source DICOM plug‐in for perfusion analysis in Horos (www.horosproject.org) [35]. First‐order moment (FOM) was calculated using an in‐house script implemented in MATLAB R2020a (MathWorks, Natick, MA, USA).

Regional analyses were conducted at both oxygen concentrations dividing lung lobes into; R1—right cranial lobe, R2—right middle lobe, R3—right caudal lobe, R4—right accessory lobe, L1—left cranial lobe, L2—left caudal lobe as described in literature [19]. An overview of a porcine lung segmented into lobes is shown in Figure S1.

### 2.7 Statistics

Student's paired *t*‐test compared the measurements at 40% and 100% FiO_2_. The relationship between p_a_O_2_ and RBC chemical shift and its linewidth were determined by fitting a simple linear regression. Multiple linear regressions assessed the interaction between RBC chemical shift and RBC peak linewidth in estimation of p_a_O_2_. Results are presented as mean values ± standard deviations. Regional effects of oxygen were assessed with two‐way ANOVA analysis across all animals. Statistical significance was defined as a *p* value below 0.05. Statistical analyses were performed using GraphPad Prism 8.0.0 (GraphPad Software, San Diego, CA, USA).

## 3 Results

### 3.1 Arterial Blood gas and Cardiopulmonary Measures

Arterial blood gas and cardiopulmonary measures showed an increase in p_a_O_2_ (182 ± 11 mmHg to 386 ± 32 mmHg, *p*‐value = 0.002) at 40% and 100% FiO_2_ prior to administering xenon gas (Table 1). No other significant differences were determined in arterial blood gas measures including hemoglobin oxygen saturation (sO_2_ ~100%) at both oxygen concentrations.

**TABLE 1:** Arterial blood gas and cardiopulmonary measures.

Oxygen level on ventilator		40%	100%	*p*
Blood gas				
pH		7.49 ± 0.01	7.49 ± 0.02	n.s.
pCo_2_	mmHg	44.03 ± 1.58	42.90 ± 3.15	n.s.
p_a_O_2_	mmHg	181.74 ± 11.22	386.13 ± 31.65	0.002
Oximetry				
ctHb	mmol/L	6.10 ± 0.70	5.93 ± 0.63	n.s.
Hctc	%	30.13 ± 3.45	29.30 ± 3.01	n.s.
sO_2_	%	100.08 ± 0.38	100.90 ± 0.22	n.s.
Cardiopulmonary				
Heart rate	beats/min	55.25 ± 13.65	63.25 ± 12.83	n.s.
Systolic pressure	mmHg	97.75 ± 14.97	97.00 ± 15.51	n.s.
Diastolic pressure	mmHg	45.25 ± 8.94	50.50 ± 8.56	n.s.
Mean arterial pressure	mmHg	61.75 ± 10.64	65.75 ± 6.42	n.s.
Respiration frequency	Hz	12.50 ± 0.87	13.50 ± 0.87	n.s.
Peak pressure	cmH_2_O	16.25 ± 1.79	17.25 ± 2.28	n.s.
PEEP	cmH_2_O	4.50 ± 0.50	4.50 ± 0.50	n.s.
Tidal volume	mL	427.50 ± 54.34	427.50 ± 48.88	n.s.
eCO_2_	%	5.73 ± 0.43	5.65 ± 0.38	n.s.

*Note:* Blood gas reports acidity in pH, partial pressure of carbon dioxide (pCO_2_), and arterial partial pressure of oxygen (p_a_O_2_). Oximetry measures hematocrit (Hctc), hemoglobin oxygen saturation (sO_2_), and total hemoglobin (ctHb). Cardiopulmonary measures include positive end‐expiratory pressure (PEEP), tidal volume and exhaled CO_2_ (eCO_2_). *p* values above 0.05 are reported as not significant (n.s.).

### 3.2 Ventilation Defect Percentage

**FIGURE 4:**
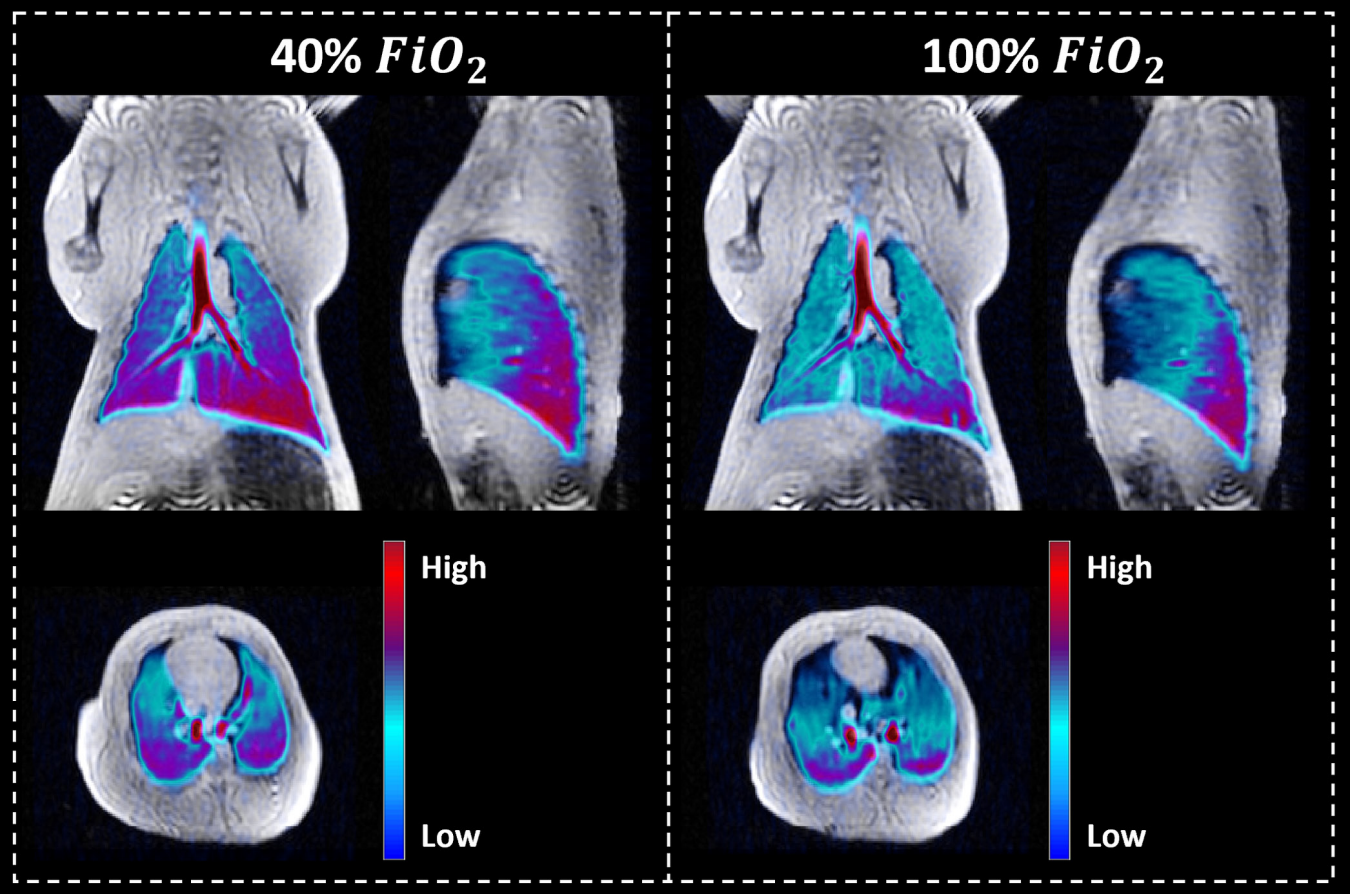
Ventilation images at 40% (left) and 100% (right) fractional inspired oxygen levels (FiO_2_). Lung volume T_1_‐weigthed ^1^H images are shown with a corresponding ^129^Xe ventilation image overlay used to calculate ventilation defect percentage.

Combined ventilation and T_1_w images are shown in Figure 4 in three orthogonal orientations with an overview of all slices in the coronal plane in Figure S1. The VDP showed no significant difference between the two oxygen concentrations (*p* value = 0.52). VDP values at 40% and 100% FiO_2_ were 6.90% ± 3.90% and 5.02% ± 4.34%, respectively.

### 3.3 Ratio, SNR, and Linewidth of Dissolved Phases and Gas

**FIGURE 5:**
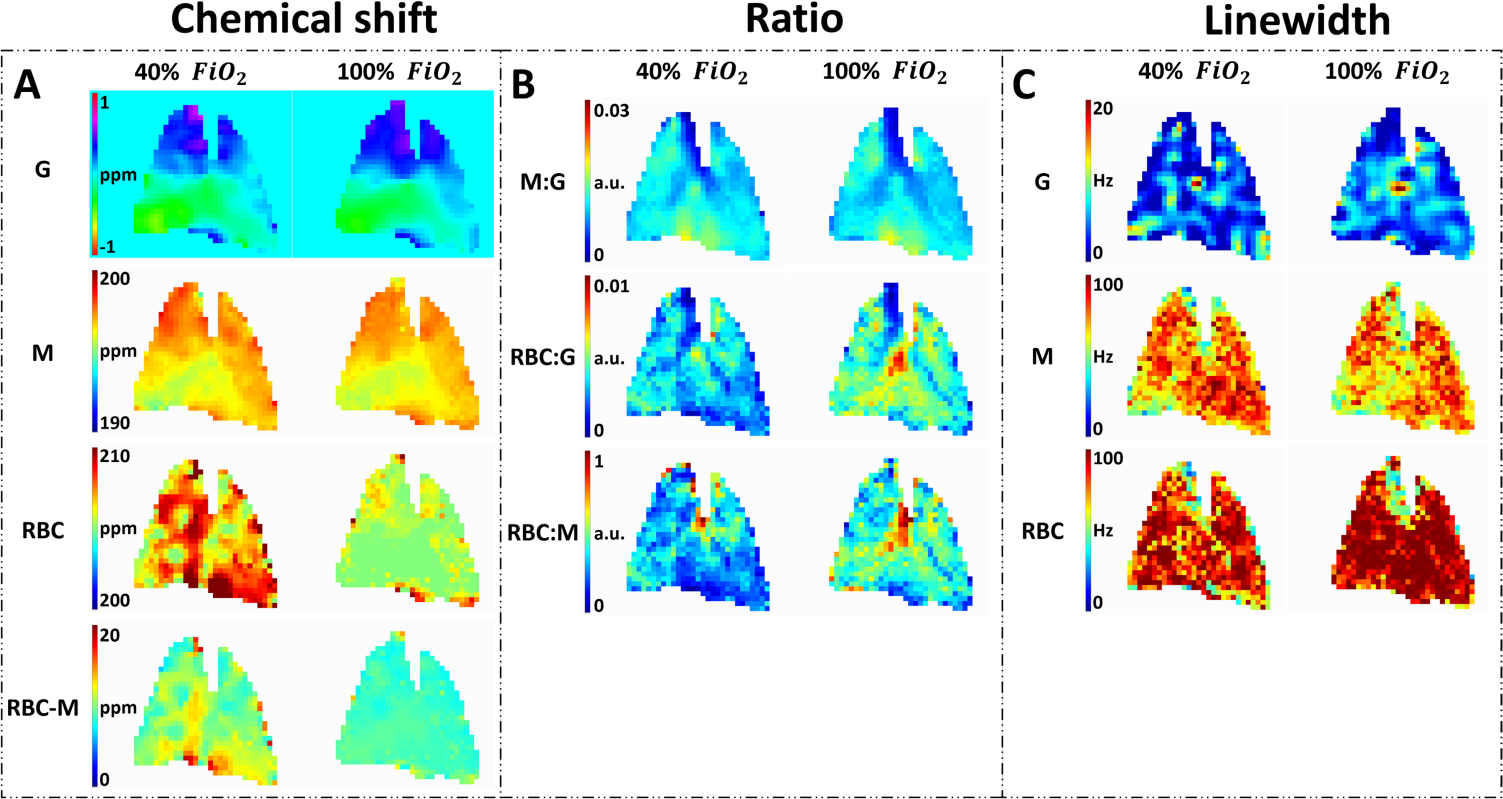
Dissolved‐phase MRSI quantified with AMARES fitting into ratios, chemical shift, and linewidth acquired at 40% (first column) and 100% (second column) inspired oxygen levels (FiO_2_). (A) Chemical shift images of gas, membrane, RBC and the difference between RBC and membrane. (B) Ratios of dissolved‐phase gas, membrane, and RBC signal ratios. (C) Linewidth of gas, membrane, and RBC. Images have been masked based on 0.6 times the gas signal mean value. Only the central slice is shown. AMARES = advanced method for accurate, robust, and efficient spectral, a.u. = arbitrary units, G = gas, M = membrane, RBC = red blood cells.

**FIGURE 6:**
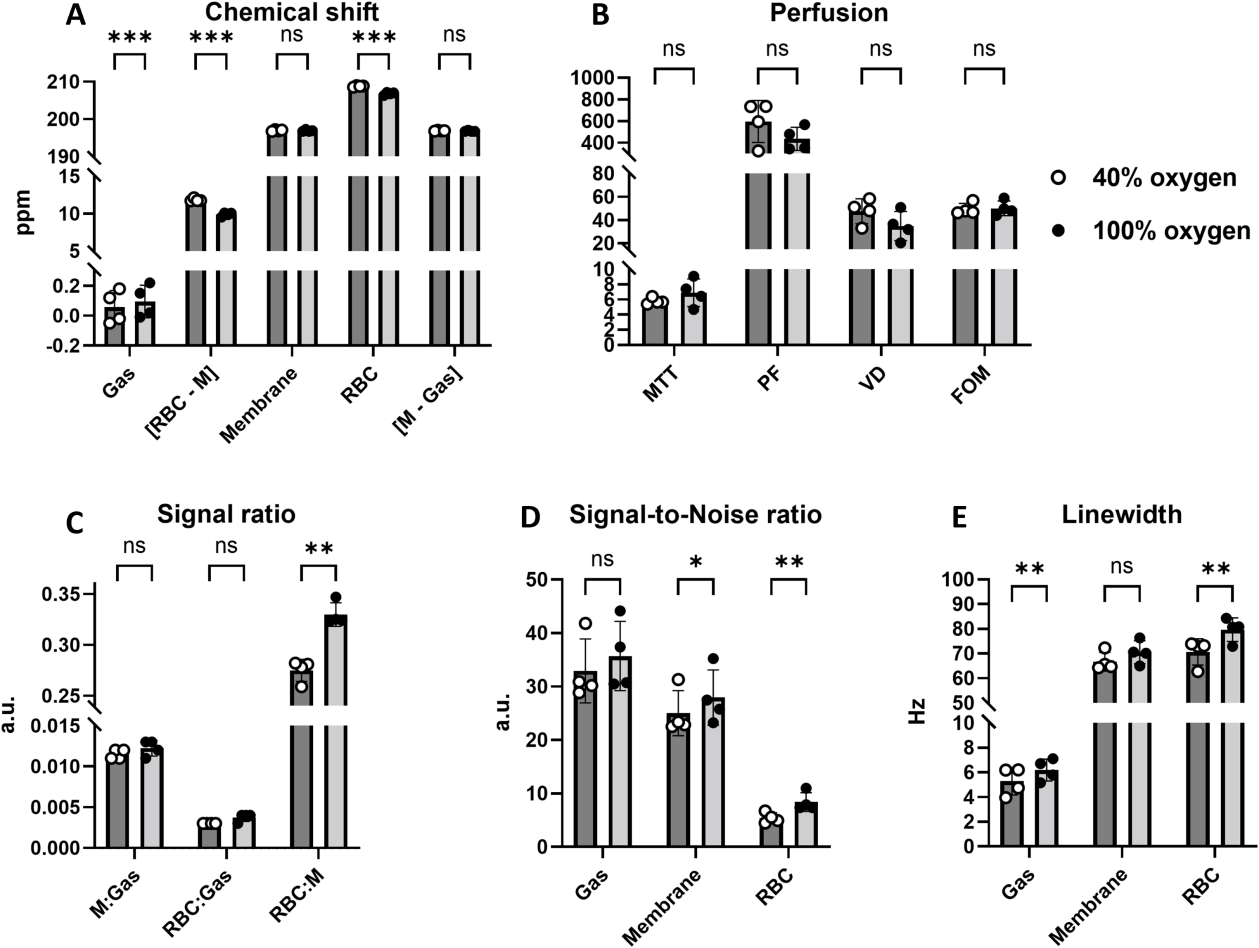
Chemical shift values (A), ratios (C), signal‐to‐noise ratios (D) and linewidths (E) of gas and dissolved phases and perfusion (B) at 40% and 100% oxygen levels. Perfusion measures include mean transit time (MTT, s), plasma flow (PF, mL/100 mL/min), volume of distribution (VD, mL/100 mL), and first‐order moment (FOM, ms). *p value < 0.05, **p value < 0.01, ***p value < 0.001, a.u. = arbitrary units, M = membrane, ns = nonsignificant, RBC = red blood cells.

Dissolved phases and gas image ratios and linewidths of the central slice are shown in Figure 5B,C. The images had a mean SNR of gas, 33 ± 6; membrane, 25 ± 4; and RBC, 5 ± 1 at 40%; and gas, 36 ± 6; membrane, 28 ± 5; and RBC, 8 ± 2 at 100% oxygen concentration (Figure 6D). There were no significant SNR differences in gas, but there were significant differences in membrane (*p* value = 0.044) and RBC (*p* value = 0.007). Ratios of gas, membrane (M), and RBC signal showed minor gas‐related changes at FiO_2_ of 40% (M:Gas: 0.012 ± 0.001, RBC:Gas: 0.003 ± 0.000) and 100% (M:Gas: 0.012 ± 0.001, RBC:Gas: 0.004 ± 0.000). However, no significant difference in RBC:Gas (*p* value = 0.06) and M:Gas ratio (*p* value = 0.06). RBC:M ratio increased significantly with the higher FiO_2_ (0.275 ± 0.011 vs. 0.330 ± 0.012, *p* value = 0.003) Ratios are illustrated in Figure 6C. Linewidth of the fitted spectra (Figure 5C) showed a significant increase in gas (5 ± 1 Hz to 6 ± 1 Hz, *p* value = 0.008) and RBC (71 ± 5 Hz to 80 ± 5 Hz, *p* value = 0.003), but no significant difference in membrane (67 ± 4 Hz to 71 ± 5 Hz) when increasing FiO_2_ from 40% to 100% (Figure 6E). Dissolved‐phase ratio and linewidth images of all coronal slices are shown in Figures S2 and S3.

### 3.4 Chemical Shift Difference of Dissolved Phases and Gas

Here 0 ppm refers to the center frequency of ^129^Xe gas‐phase peak. Chemical shift values were as follows: gas, 0.06 ± 0.11 ppm; membrane, 196.97 ± 0.23 ppm; RBC, 208.80 ± 0.17 ppm at 40%; and gas, 0.10 ± 0.11 ppm; membrane, 196.91 ± 0.24 ppm; RBC, 206.81 ± 0.36 ppm at 100% oxygen concentration. Results are illustrated in the bar plot of Figure 6A with images of the central slice in Figure 5A. In porcine lungs, FiO_2_ of 40% resulted in a higher chemical shift difference of RBC and membrane (11.84 ± 0.19 ppm) when compared with 100% (9.89 ± 0.26 ppm), with a *p* value below 0.001. The difference was mainly observed as local differences in the posterior part of the lungs, as shown in Figure S4. Gas chemical shifts also showed a significant difference between the two oxygen concentrations (*p* value < 0.001). Nevertheless, when adjusted to dissolved‐phase membrane chemical shift, the difference became nonsignificant. Chemical shift differences in the anterior part of the lungs were comparable for both oxygen concentrations.

### 3.5 Partial Pressure of Arterial Oxygen Maps

**FIGURE 7:**
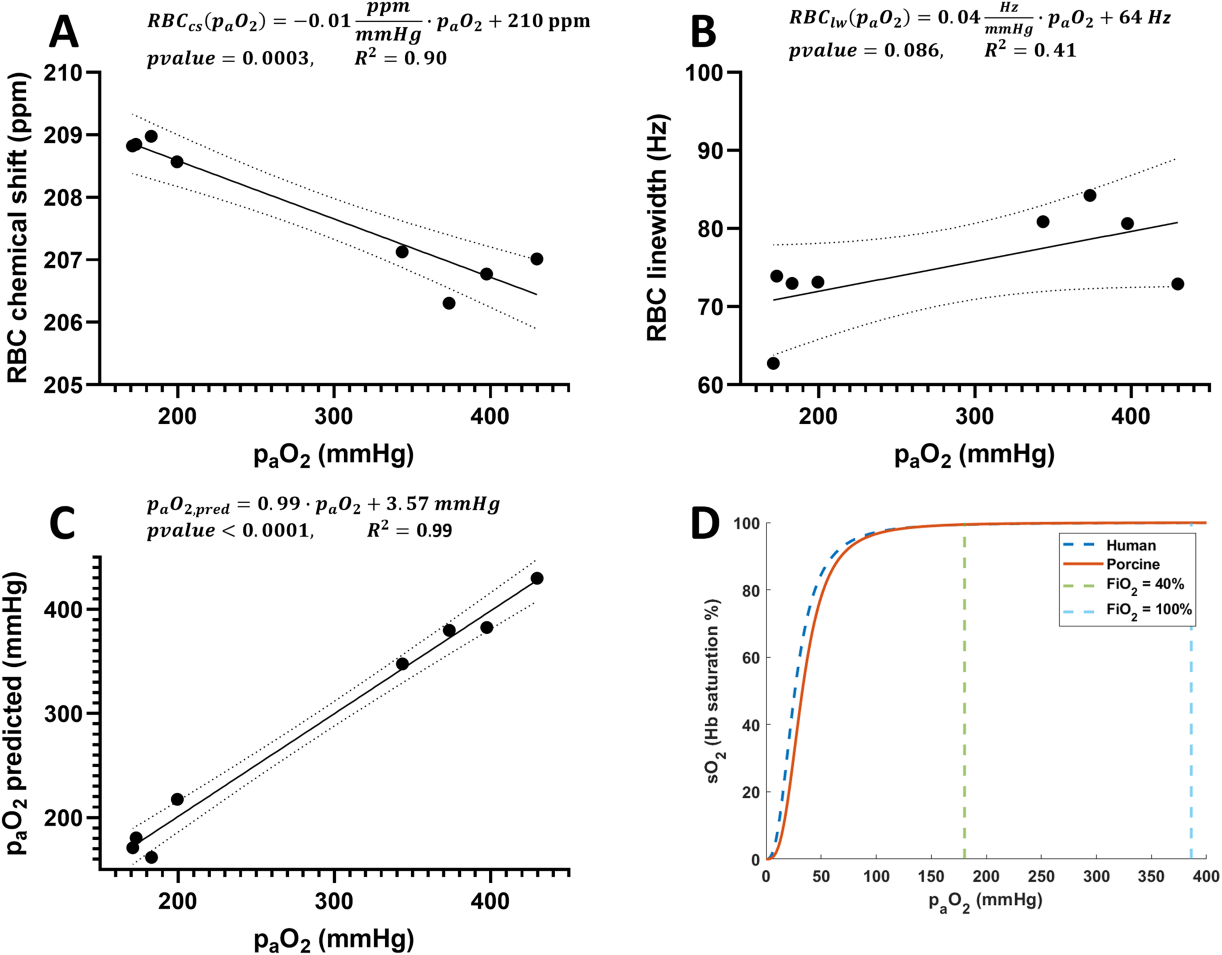
Simple linear regression fit between p_a_O_2_ and RBC chemical shift (A) and RBC spectra linewidth (B). The correlation between predicted and measured p_a_O_2_ as a multiple linear regression including RBC chemical shift and linewidth (C). Human and porcine oxygen‐hemoglobin dissociation curve (D) based on previously published models [23] with indication of p_a_O_2_ at FiO_2_ = 40% and 100%. FiO_2_ = fractional inspired oxygen levels, Hb = hemoglobin, hemoglobin oxygen saturation (sO_2_), p_a_O_2_ = arterial oxygen partial pressure, RBC = red blood cells, RBC_cs_ = dissolved‐phase RBC chemical shift, RBC_lw_ = dissolved‐phase RBC linewidth.

Simple linear regression fit between p_a_O_2,_ and RBC chemical shift or RBC spectra linewidth showed a strong correlation between chemical shift and p_a_O_2_ (Figure 7A: *R* ^2^ = 0.90, *p* value = 0.0003) while the linewidth did not reach statistically significance (Figure 7B: *R* ^2^ = 0.41, *p* value = 0.086). The linear regression models can be described by the following equations:

(2) $${\mathrm{RBC}}_{\mathrm{cs}}\left(p_{a}O_{2}\right)=-0.01\;\frac{\mathrm{ppm}}{\text{mmHg}}\cdot p_{a}O_{2}+210\;\mathrm{ppm},$$

(3) $${\mathrm{RBC}}_{\mathrm{lw}}\left(p_{a}O_{2}\right)=0.04\;\frac{\mathrm{Hz}}{\text{mmHg}}\cdot p_{a}O_{2}+64\;\mathrm{Hz},$$

Changing the equation's linear dependence from p_a_O_2_ to chemical shift and linewidth, p_a_O_2_ maps can be estimated:

(4) $$p_{a}O_{2}\left(\mathrm{cs}\right)=-100\;\frac{\text{mmHg}}{\mathrm{ppm}}\cdot {\mathrm{RBC}}_{\mathrm{cs}}+21,000\;\mathrm{mmH}$$

(5) $$p_{a}O_{2}\left(\mathrm{lw}\right)=25\;\frac{\text{mmHg}}{\mathrm{Hz}}\cdot {\mathrm{RBC}}_{\mathrm{lw}}-1600\;\text{mmHg}$$

In Equations (4) and (5), RBC_cs_ is RBC chemical shift in ppm and RBC_lw_ is the linewidth of the RBC peak in hertz. Extending the simple linear regression model to include both chemical shift (cs), linewidth (lw), and their interaction term (cs·lw) produces a multiple regression linear model with a stronger correlation to p_a_O_2_ (*R* ^2^ = 0.97, *p* value < 0.001):

(6) $$\begin{array}{c} p_{a}O_{2}\left(\mathrm{cs},\mathrm{lw}\right)=-546\;\frac{\text{mmHg}}{\mathrm{ppm}}\cdot {\mathrm{RBC}}_{\mathrm{cs}}-1171\;\frac{\text{mmHg}}{\mathrm{Hz}}\cdot {\mathrm{RBC}}_{\mathrm{lw}}\\ \;+6\;\frac{\text{mmHg}}{\mathrm{ppm}\cdot \mathrm{Hz}}\cdot {\mathrm{RBC}}_{\mathrm{cs}\cdot \mathrm{lw}}+114,067\;\text{mmHg}, \end{array}$$

The multiple linear regression model indicates that each component, RBC chemical shift (*p* value = 0.0097), the intercept (*p* value = 0.0096), the linewidth (*p* value = 0.018), and the interaction term (*p* value = 0.018) has significance in predicting p_a_O_2_.

The linear regression models (Equations 2 and (3)) and the correlation between measured p_a_O_2_ and predicted p_a_O_2_ (Equation 6) are shown in Figure 7A–C. Correlations show a small deviation between predicted and measured p_a_O_2_ when including linewidth in the regression model with a slope getting closer to 1 (*R* ^2^ = 0.99) and an intercept of 3.57 mmHg.

**FIGURE 8:**
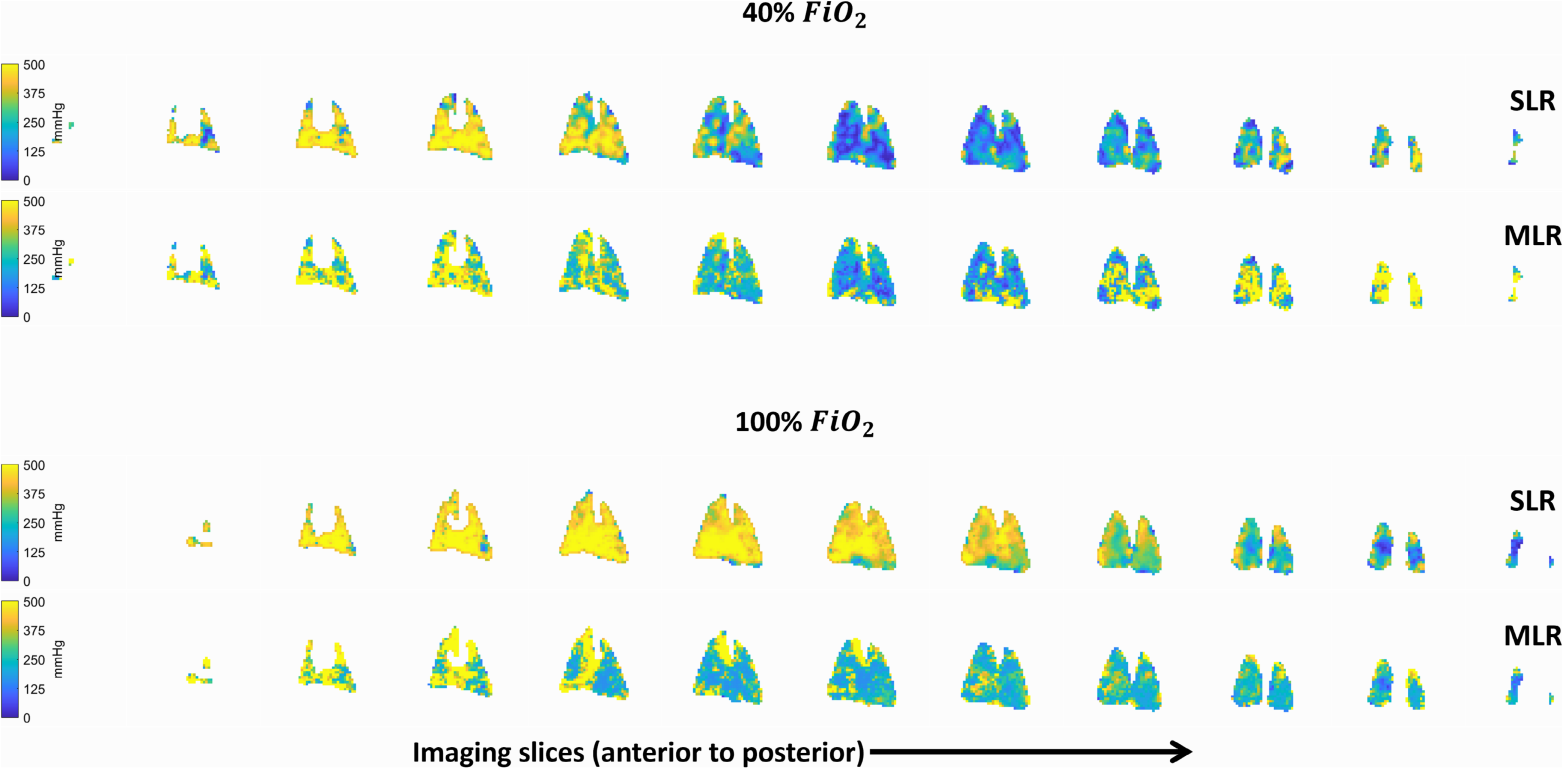
Arterial oxygen partial pressure (p_a_O_2_) maps based on simple linear regression fit (SLR) and multiple linear regression fit (MLR) between RBC chemical shift, RBC linewidth and arterial blood gas p_a_O_2_ at fractional inspired oxygen levels (FiO_2_) of 40% (top rows) and 100% (bottom rows). RBC = red blood cells.

Arterial oxygen partial pressure (p_a_O_2_) maps calculated from the simple linear regression fit of RBC chemical shift and the multiple linear regressions at FiO_2_ of 40% and 100% are shown in Figure 8.

### 3.6 Perfusion Quantification

**FIGURE 9:**
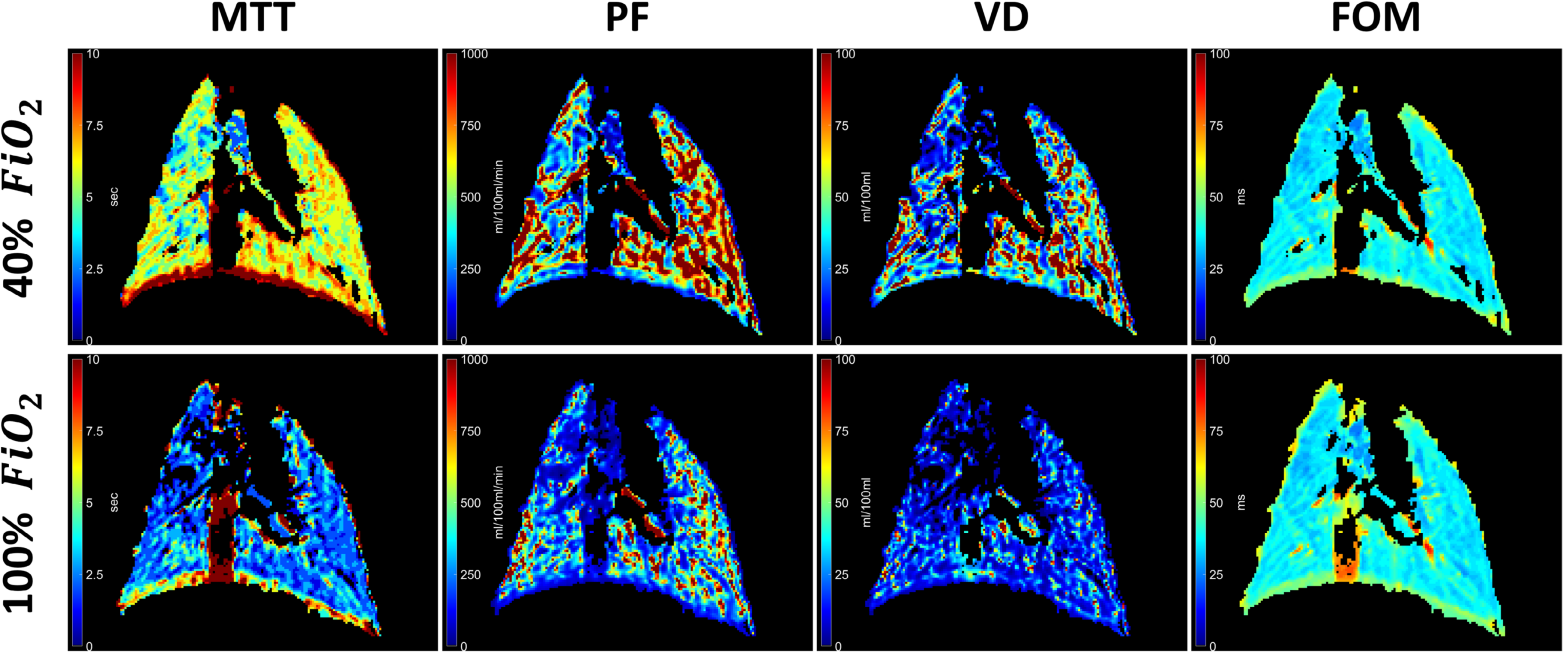
Perfusion images calculated from the TRICKS T_1_w DCE images masked to the lung. Calculated images include mean transit time (MTT), plasma flow (PF), volume of distribution (VD), and first‐order moment (FOM) acquired at fractional inspired oxygen level (FiO_2_) of 40% (top row) and 100% (bottom rows). Only the central slice is shown. DCE = dynamic contrast enhanced, T_1_w = T_1_ weighted, TRICKS = time‐resolved imaging of contrast kinetics.

Whole lung perfusion indicated no significant changes between the two oxygen concentrations (Figures 6B and 9). Perfusion is evaluated in MTT (5.78 ± 0.42 s vs. 6.90 ± 1.83 s, *p* value = 0.33), plasma flow (596 ± 194 mL/100 mL/min vs. 435 ± 107 mL/100 mL/min, *p* value = 0.15), volume of distribution (48 ± 11 mL/100 mL vs. 35 ± 13 mL/100 mL, *p* value = 0.26), and the FOM (49 ± 5 ms vs. 50 ± 6 ms, *p* value = 0.48). Perfusion images of all coronal slices are shown in Figure S5.

### 3.7 Regional Analysis of Dissolved Phase Metrics and Perfusion

**FIGURE 10:**
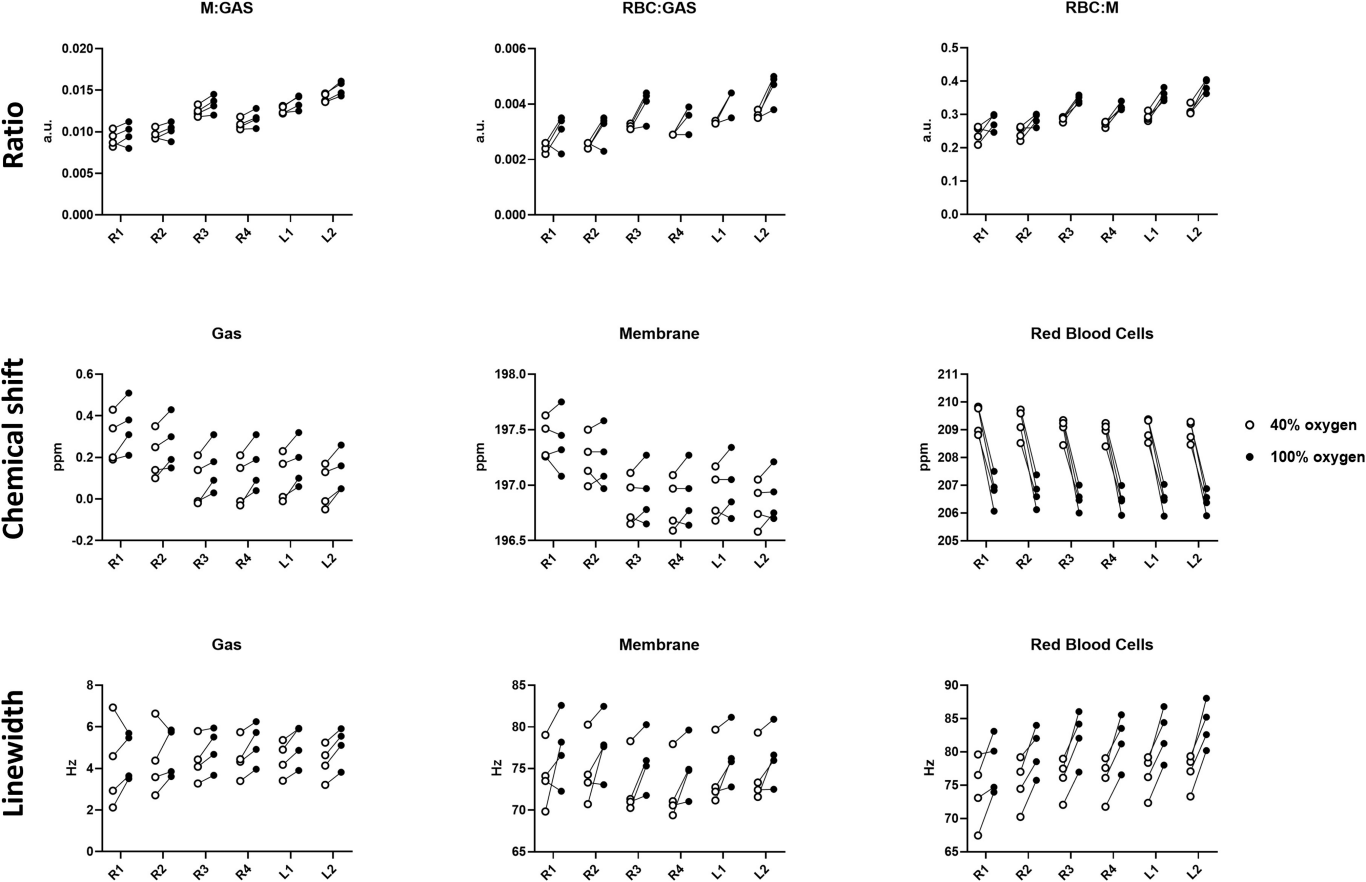
Regional dissolved‐phase ratios, chemical shifts, and linewidth at fractional inspired oxygen levels (FiO_2_) of 40% (white circle) and 100% (black circle). Two‐way ANOVA results are shown in Table 2.

**FIGURE 11:**
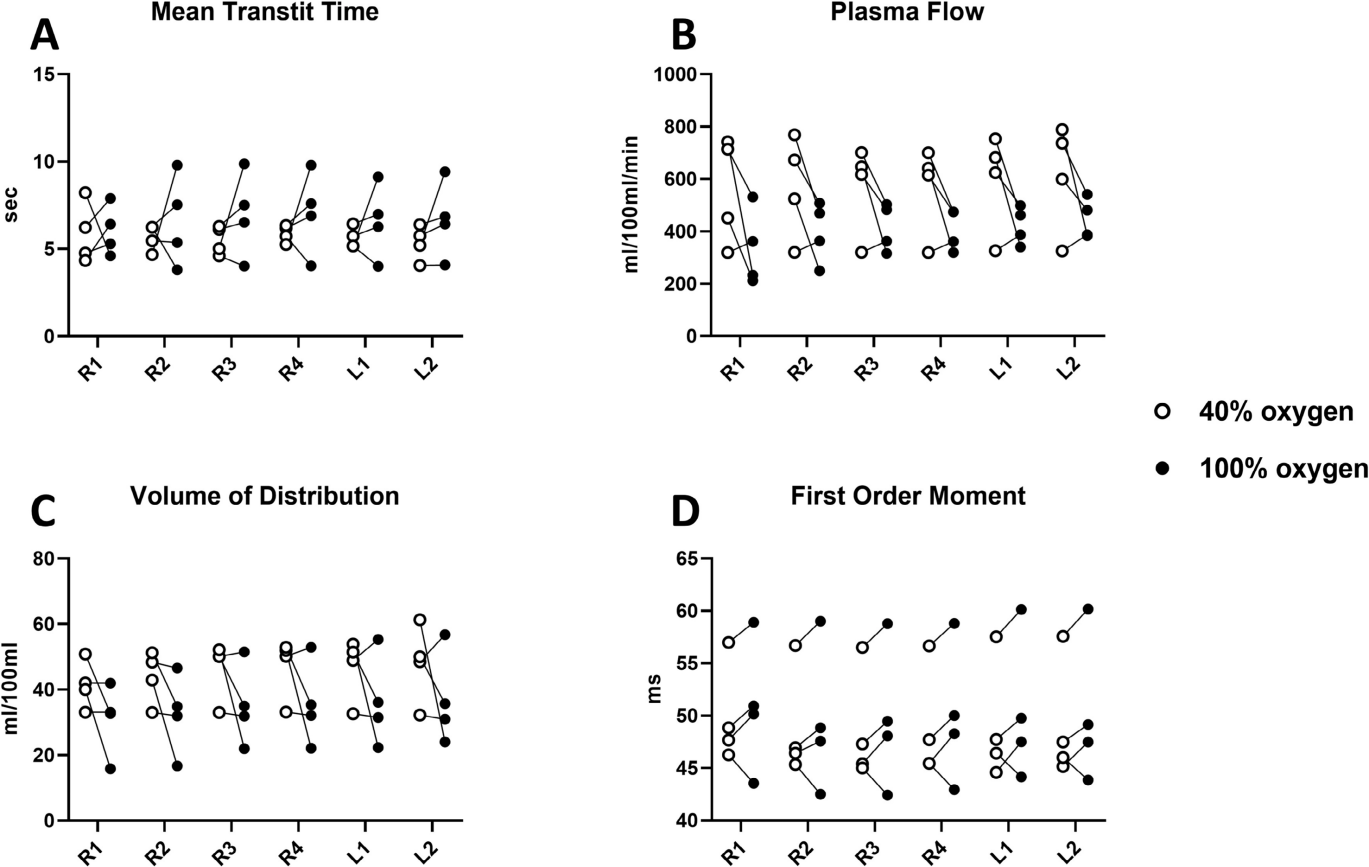
Regional perfusion at fractional inspired oxygen levels (FiO_2_) of 40% (white circle) and 100% (black circle). (A) Mean transit time, (B) plasma flow, (C) volume of distribution, and (D) first‐order moment. Two‐way ANOVA results are shown in Table 2.

Regional variation at FiO_2_ of 40% and 100% of dissolved‐phase ratios, chemical shifts, and linewidths are plotted in Figure 10, with corresponding perfusion measures in Figure 11. Two‐way ANOVA results (Tables 2 and S1) indicate increased dissolved‐phase ratios, chemical shifts, and linewidths across regions, which was not observed in perfusion imaging. Increasing oxygen showed an increase in gas chemical shift and RBC:M ratio with a decrease in RBC chemical shift. Combined effects of regions and oxygen concentration were found in M:Gas, RBC:Gas, membrane chemical shift, and RBC linewidth. Results indicate that lung regions are affected differently accordingly to oxygen concentration.

**TABLE 2:** Two‐way ANOVA of regional effects at two oxygen concentrations in DCE perfusion and dissolved‐phase ratios, chemical shifts, and linewidth. Region x Oxygen is the interaction between the two variables.

	Region	Oxygen	Region × oxygen
M:Gas	< 0.0001	0.06	0.02
RBC:Gas	< 0.0001	0.06	0.01
RBC:M	0.001	0.003	0.23
GAS CS	< 0.0001	0.03	0.31
MEM CS	< 0.0001	0.50	0.01
RBC CS	0.01	0.001	0.70
GAS LW	0.48	0.09	0.87
MEM LW	< 0.0001	0.10	0.77
RBC LW	< 0.0001	0.008	0.01
MTT	0.93	0.45	0.40
PF	0.41	0.13	0.81
VD	0.19	0.24	0.84
FOM	0.28	0.44	0.21

Abbreviations: DCE = dynamic contrast enhanced, M = membrane, RBC = red blood cells, CS = chemical shift, LW = line width, MTT = mean transit time, PF = plasma flow, VD = volume of distribution, FOM = first order moment.

## 4 Discussion

In this study, a 3D Cartesian MRSI sequence for dissolved‐phase and gas imaging detected decreased chemical shift changes in healthy porcine lungs with increased fractional inspired oxygen (FiO_2_). Significant changes were observed in the RBC chemical shift (decrease) and RBC:M ratio (increase) at FiO_2_ of 40% and 100%. RBC chemical shift showed a strong correlation to arterial oxygen partial pressure (p_a_O_2_) from arterial blood gas suggesting a direct measurement of regional variations of p_a_O_2_ using dissolved‐phase RBC chemical shift imaging. Our data confirm that the chemical shift information is a better predictor for p_a_O_2_ than perfusion metrics and would often suffice, but the fact that the linewidth is associated with p_a_O_2_ and thus the chemical shift information supports the use of the interaction term in modeling of p_a_O_2_ estimations. Lung lobe analyses indicate that lung regions are affected differently according to the oxygen concentration in dissolved‐phase ratios, chemical shifts, and linewidths, which was not observed in perfusion imaging.

Mechanisms of the ^129^Xe chemical shift dependence on blood oxygenation are not fully understood, with the main factor thought to be conformational changes in the hemoglobin molecule during oxygen binding [13, 24]. Norquay and Wolber evaluated changes in the ^129^Xe RBC chemical shift as a function of blood oxygenation in human whole blood samples in vitro, using oxygen saturation (sO₂) levels ranging from 1.00 to below 0.10 [13, 24]. Results showed that RBC chemical shift was nonlinearly dependent on the measured sO_2_ and could be described as an exponential function:

(7) $$\delta \left(sO_{2}\right)=\alpha \exp\left(\mathrm{\beta s}O_{2}\right)+\delta_{0},$$

(8) $$\delta \left(sO_{2}\right)=9.3\cdot 10^{-4}\cdot \exp\left(8.62\cdot sO_{2}\right)+20.4\;\left[\mathrm{ppm}\right],$$

In the porcine model, FiO_2_ is significantly higher than ambient air (21% vs. 40% and 100%), and the exponential model does not account for hyperoxia as the samples included were from normoxia to hypoxia. Including normoxia and hypoxia would have been a valuable addition to our study providing additional insights to the hypoxic effects on dissolved‐phase chemical shifts. Because arterial blood gas measures of sO_2_ were not affected at 40% oxygen, the local veterinarian has approved to use FiO_2_ of 15% and 21% for future studies.

**FIGURE 12:**
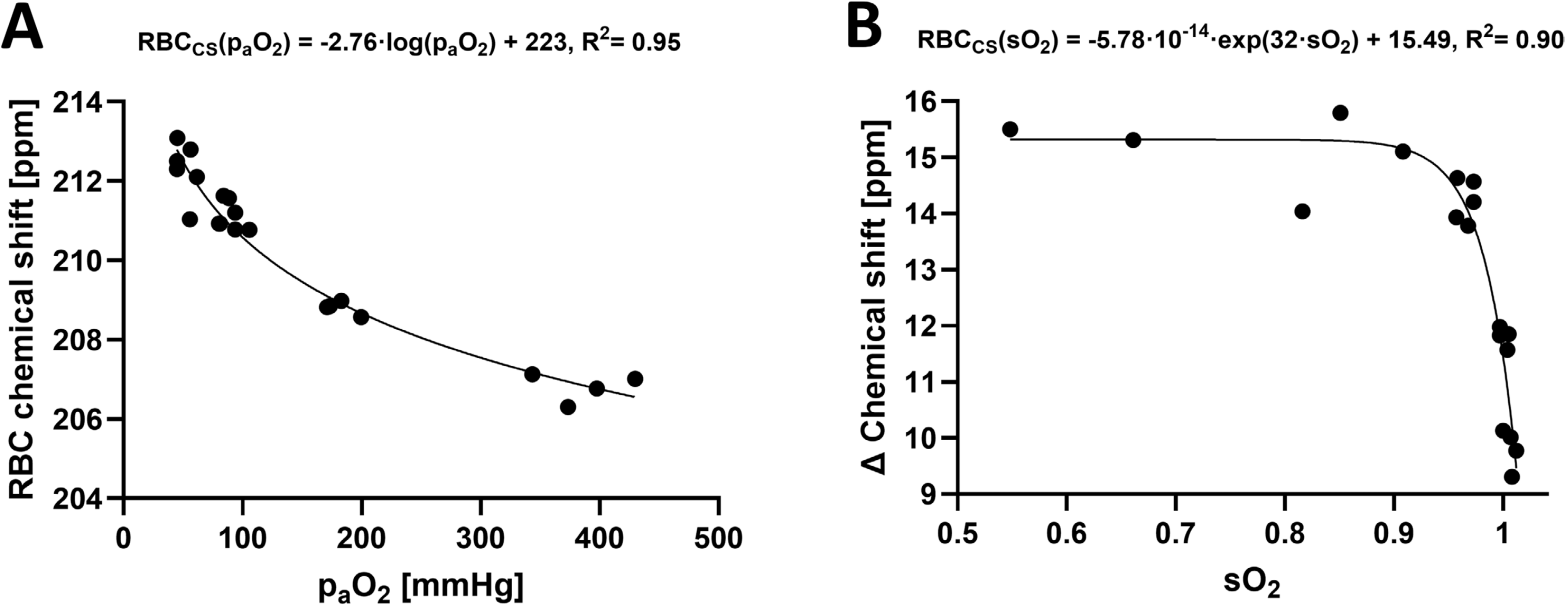
RBC chemical shift according to oxygenation. (A) Chemical shift of red blood cell (RBC) signal from increased partial pressure of oxygen (p_a_O_2_) fitted with a logarithmic regression model. (B) Delta chemical shift of membrane and RBC from increasing hemoglobin oxygen saturation (sO_2_), from hypoxia to hyperoxia, fitted with an exponential model.

Studies with fully anesthetized pigs are typically performed at a FiO_2_ of 40%, as anesthetized animals on a mechanical ventilator may experience a drop in oxygen saturation. This is also the case in humans during surgery with high levels of anesthesia. Therefore, FiO_2_ of 21% is expected to cause a lower saturation when anesthetized as to being awake as shown in Figure 12B (sO_2_ ~0.96). Including additional data, we find that the RBC chemical shift relationship in pigs with FiO_2_ of 15%, 21%, 40%, and 100% (p_a_O_2_ = 45 to 430 [mmHg]; sO_2_ = 0.55 to 1.00) can be described with a logarithmic (Figure 12A) or exponential (Figure 12B) model. Inclusion of lower FiO_2_ supports the findings of decreased RBC chemical shift with higher blood oxygenation. The additional data indicate a logarithmic relationship between RBC chemical shift and p_a_O_2_. Our study applied a linear regression model; nevertheless, when evaluating the range from FiO_2_ at 40% to 100% it is approximately linear.

Chemical shift of RBC, in human whole blood exhibited an exponential growth model when increasing sO_2_ from hypoxia to normoxia (FiO_2_ = 21%). In the porcine model, we found that the RBC chemical shift decreased nonlinearly as a function of sO_2_, which is opposite the previous observations in human blood [13, 24] but is consistent with observations reported in rats [36]. Second, the chemical shifts of rats and pigs at normoxia is 211 ppm in both species—a value notable below the 218 ppm reported in humans. Examining the fitted exponential model in Figure 12B, the growth factor is approximately four times higher in pigs (32) when compared with humans (8.6). The growth factor may be related to the relationship between p_a_O_2_ and sO_2_ described in the ODC which primarily describes changes in sO_2_ (oxygen binding) when p_a_O_2_ drops below baseline resting state conditions (~100 mmHg at 21% oxygen). Increasing p_a_O_2_ above resting state conditions does not affect hemoglobin binding as the sO_2_ in the ODC has reached saturation [37]. There are differences in human and porcine ODCs [23]; nevertheless, at p_a_O_2_ above 150 mmHg, as evaluated in this study, the curves are similar (Figure 7D).

The mechanisms driving the RBC chemical shift change in humans and animals as a function of oxygenation is currently not well understood but is expected to be related to Hb‐specific xenon–protein interactions [38, 39] during oxygen binding. Porcine hemoglobin is different from human hemoglobin [23], with altered functional properties and electrostatic interactions modulating O_2_ affinity [40, 41]. Electrostatic interactions influence hemoglobin binding to the cytoplasmic surface of the RBC membrane [42] which could induce a shielding effect causing the opposite RBC chemical shift response to oxygen concentration in pigs when compared with human blood. Previous animal studies have shown that hemoglobin concentrations and HCTs decrease during anesthetics [43]. Nevertheless, no change was found in hemoglobin concentrations or HCTs from pig blood gas measurements (Table 1). Bulk magnetic susceptibility (BMS) may be a contributing factor as shown by increased RBC linewidth at the higher oxygen concentration [44]. BMS affects regional ^129^Xe signal intensities and chemical shifts in the gas and RBC phase [45]. This phenomenon (chemical shifts and magnetic susceptibility caused by a paramagnetic compound) is supported by results of dissolved‐phase ratios, chemical shifts, and linewidth regional differences, which was not observed in perfusion imaging. Wolber et al. predicted that intracellular susceptibility effects should cause only a 0.25‐ppm shift, in humans, making it unlikely to be a major factor in the observed > 5 ppm decrease from 0.5 to 1.00 sO_2_ [24]. Gas linewidth increased when evaluating the whole lung; however, at regional levels, no significant effects were observed as a result of increased oxygen concentration. As effects of oxygen was mainly observed in RBC (RBC:M, chemical shift and linewidth), it is considered most oxygen sensitive. The RBC linewidth observation was included in the linear regression analysis of p_a_O_2_, and even though it did not correlate significantly to p_a_O_2_ in the simple model, it did improve the fit of the multiple linear regression model when including the interaction with RBC chemical shift. The additional information in the interaction is supported by the chemical shift and linewidth regional effects of oxygen concentration varying across lung regions (Table 2). This is unexplored, and an accurate description and measurement of porcine dissolved‐phase changes according to oxygen concentration is important as pig physiology resembles humans the most and are frequently used for experimental and physiological studies (hemodilution, systemic hypoxia, ischemia–reperfusion, or resuscitation), as such highlighting the necessity of future studies to model the differences between porcine and human oxygen effects on ^129^Xe dissolved‐phase RBC from hypoxia to hyperoxia. The prospective study could include in vitro whole blood measurements as per Norquay et al. [13] for both species with evaluation of the chemical shift alteration in the range of FiO_2_ from 15% to 100% combined with measures of sO_2_ from 10% to 100%. The study would provide new insights to oxygen‐related changes in human blood at FiO_2_ above 21% and provide an accurate conversion model between species.

Dissolved‐phase signal ratios of M:Gas and RBC:M were comparable with findings in humans [11], whereas RBC:Gas was lower. RBC:M is reported to be the most reproducible measurement [46] as RBC:Gas and M:Gas being highly sensitive to lung inflation effects [45, 46, 47, 48]. In our study, we increased FiO_2_ from 40% to 100% with a saturation period of 20 min, keeping lung volume and other mechanical ventilation parameters fixed. Effects on xenon gas exchange ratios are then restricted to the oxygen concentration at the alveolar level. Gas and RBC signal T1 relaxation have reverse effects with increased oxygen concentration. The gas phase is sensitive to paramagnetic oxygen, shortening the T1 relaxation time with a decay rate proportional to the oxygen concentration [3, 25]. Acquired dissolved‐phase signal exponential decay supports the decrease in gas relaxation time from 14.10 ± 1.27 ms to 9.08 ± 0.94 ms as FiO_2_ is increased from 40% to 100% (FID data example in Figure S6). A potential hypothesis for the observed increase in RBC:M is that the T1 relaxation time of RBCs lengthens with increasing blood oxygenation [13, 49], enhancing the detectable signal without altering perfusion. Furthermore, regional variations in alveolar microstructure, capillary blood volume, and blood flow dynamics may also contribute to the differential responses observed [50]. Therefore, we performed regional analysis of the segmented lung lobes combined with a two‐way ANOVA (Figures 10 and 11, Tables 2 and S1). The analyses showed a combined effect of oxygen and regions indicating that lung regions are affected differently according to oxygen concentration. These findings support the use of regional information in evaluation of pulmonary physiology. These differences may reflect variations in ventilation–perfusion matching across lung regions, as posterior lung regions are often better perfused and more affected by oxygen concentration changes due to gravitational effects. Future studies could investigate whether these variations are driven by differences in local oxygen delivery or uptake, providing deeper insight into the physiological mechanisms underlying regional oxygenation patterns.

Ventilation images showed no significant differences in VDP between the two oxygen concentrations. This is expected in healthy porcine subjects because ventilation should not be affected by hyperoxia. Dynamic contrast‐enhanced MRI provided lung perfusion imaging [51], where several perfusion metrics (MTT, PF, VD, and FOM) were calculated to measure changes with increased oxygen. Nevertheless, no significant differences in pulmonary perfusion were determined, at most, a trend in plasma flow. Oxygen is a potent vasodilator lowering pulmonary vascular resistance, but the effects are comparable at FiO_2_ of 40% or above [52, 53]. And our results could indicate unchanged pulmonary vascular resistance between oxygen concentrations.

Although the study shows promising results, one limitation is the number of pigs (4) and fractional inspired oxygen levels (2). Including more oxygen levels would provide a stronger predictive value and determine the optimal model to describe the relationship between p_a_O_2_ and RBC chemical shift. Furthermore, having results from hypoxic and normal atmospheric conditions (21% oxygen) in pig and human could link our findings to literature and clinical examinations. The effect of anesthetics [43] and laying on the table should also be considered in the use of animal models; future studies should randomize the order of oxygen administration. Nevertheless, no significant differences were measured in HCT and hemoglobin at 40% and 100% FiO_2_, indicating minimal effects.

As future perspectives, the effects of xenon gas chemical shift alteration in whole blood of humans and pigs should be explored. The study would provide new insights to oxygen‐related changes in human blood at FiO_2_ above 21% and provide an accurate conversion model between species. These findings could have significant relevance for studying and diagnosing diseases such as COPD, IPF, or pulmonary hypertension, where regional oxygenation and gas exchange are disrupted. Additionally, the ability to noninvasively map regional oxygenation could aid in presurgical assessments for lung cancer patients or monitoring the progression of hypoxia‐related conditions. Including intervention or disease models (e.g., pulmonary embolism, presurgical screening in COPD, or lung cancer) would have an increased clinical translation to evaluate the sensitivity to physiological pulmonary effects. For clinical translation, the MRSI data provided images of CRLB of gas, membrane, and RBC from the AMARES quantification. CRLB could serve as indicators of the dissolved‐phase image quality and robustness; however, they were not explored in this study. SNR measures showed sufficient signal for clinical evaluation at improved resolution when compared with the literature [7, 11, 12, 54]. This supports the idea of applying undersampling to trade SNR for faster acquisition time [55]. Faster acquisition time may be utilized for either higher resolution or shorter breath‐hold. The latter being useful for patient comfort and for patients with reduced lung function.

## 5 Conclusions

Dissolved‐phase ^129^Xe chemical shift in RBCs was found to decrease linearly with arterial oxygen partial pressure (p_a_O_2_) in healthy porcine lungs at inspired oxygen levels of 40% and 100% in a single breath‐hold. In healthy porcine lungs, we detect decreased regional RBC chemical shift, increased RBC to membrane ratio, and RBC linewidth with increased oxygen saturation sO_2_. Our data suggest that regional p_a_O_2_ prediction is possible with a multiple linear regression model including RBC chemical shift and linewidth as the combined effect of oxygen affects lung regions differently. This work represents an important step toward translating dissolved‐phase hyperpolarized ^129^Xe imaging into clinical settings for improved evaluation of pulmonary physiology and oxygenation dynamics.

## Conflicts of Interest

M.V. and R.F.S. are employees of GE HealthCare. The authors report no conflicts of interest.

## Supporting information


**Figure S1** Ventilation images at oxygen levels of 40% (top row) and 100% (middle row). T_1_‐weigthed ^1^H MRI and ^129^Xe ventilation image overlay is shown in the top left which was used to calculate ventilation defect percentage. Regional segmentation mask of the animal lung lobes (R1‐R4 and L1‐L2) is shown in the top right. Corresponding image slices for segmentation, 40% and 100% oxygen concentrations are indicated with a red border.
**Figure S2.** Dissolved phase images of gas, membrane, and RBC with ratios acquired at fractional inspired oxygen levels (FiO_2_) of 40% (top six rows) and 100% (bottom six rows). M:G = membrane/gas, RBC:G = RBC/gas, RBC:M = RBC/membrane. Images have been masked based on the gas signal. G = gas, M = membrane, RBC = red blood cells.
**Figure S3.** Linewidth of gas, membrane and RBC peaks determined from the AMARES quantification at fractional inspired oxygen levels (FiO_2_) of 40% (top 3 rows) and 100% (bottom 3 rows). AMARES = Advanced Method for Accurate, Robust, and Efficient Spectral. M = membrane, RBC = red blood cells.
**Figure S4.** Chemical shift images of gas, membrane, and RBC at fractional inspired oxygen levels (FiO_2_) 40% (top rows) and 100% (bottom rows). The chemical shift difference between RBC and membrane is shown at the last row in each oxygen section. M = membrane, RBC = red blood cells.
**Figure S5.** Perfusion images calculated from the TRICKS T_1_w DCE images masked to the lung. Calculated images include mean transit time (MTT), plasma flow (PF), volume of distribution (VD) and first order moment (FOM) acquired at fractional inspired oxygen levels (FiO_2_) of 40% (top four rows) and 100% (bottom four rows). The central 10 slices of 88 are shown. DCE = dynamic contrast enhanced, T_1_w = T_1_ weighted, TRICKS = Time‐Resolved Imaging of Contrast KineticS.
**Figure S6*.*** Gas T1 exponential decay of dissolved‐phase signal at inspired oxygen levels (FiO_2_) of 40% (fit in red) and 100% (fit in blue).
***Table S1. Regional descriptive statistics of two oxygen levels in dissolved phase ratios, chemical shifts, linewidth, and perfusion.*** Fractional inspired oxygen level (FiO_2_), Membrane (M), Red blood cells (RBC), Chemical shift (CS), Linewidth (LW), Mean transit time (MTT), plasma flow (PF), volume of distribution (VD) and first order moment (FOM). R1—right cranial lobe, R2—right middle lobe, R3—right caudal lobe, R4—right accessory lobe, L1—left cranial lobe, L2—left caudal lobe.

## Data Availability

The data that support the findings of this study are available from the corresponding author upon reasonable request.
