# Supplementary material for: MR Spectroscopic Imaging of Hyperpolarized 129‐Xenon in the Dissolved‐Phase to Determine Regional Chemical Shifts of Hyperoxia in Healthy Porcine Lungs
Source: NMR Biomed. 2025 May 18;38(6):e70063. doi: 10.1002/nbm.70063 (PMC12086352; doi:10.1002/nbm.70063)
Supplement: Supplementary file 1 — Figure S1 Ventilation images at oxygen levels of 40% (top row) and 100% (middle row). T1‐weigthed 1H MRI and 129Xe ventilation image overlay is shown in the top left which was used to calculate ventilation defect percentage. Regional segmentation mask of the animal lung lobes (R1‐R4 and L1‐L2) is shown in the top right. Corresponding image slices for segmentation, 40% and 100% oxygen concentrations are indicated with a red border. Figure S2. Dissolved phase images of gas, membrane, and RBC with ratios acquired at fractional inspired oxygen levels (FiO2) of 40% (top six rows) and 100% (bottom six rows). M:G = membrane/gas, RBC:G = RBC/gas, RBC:M = RBC/membrane. Images have been masked based on the gas signal. G = gas, M = membrane, RBC = red blood cells. Figure S3. Linewidth of gas, membrane and RBC peaks determined from the AMARES quantification at fractional inspired oxygen levels (FiO2) of 40% (top 3 rows) and 100% (bottom 3 rows). AMARES = Advanced Method for Accurate, Robust, and Efficient Spectral. M = membrane, RBC = red blood cells. Figure S4. Chemical shift images of gas, membrane, and RBC at fractional inspired oxygen levels (FiO2) 40% (top rows) and 100% (bottom rows). The chemical shift difference between RBC and membrane is shown at the last row in each oxygen section. M = membrane, RBC = red blood cells. Figure S5. Perfusion images calculated from the TRICKS T1w DCE images masked to the lung. Calculated images include mean transit time (MTT), plasma flow (PF), volume of distribution (VD) and first order moment (FOM) acquired at fractional inspired oxygen levels (FiO2) of 40% (top four rows) and 100% (bottom four rows). The central 10 slices of 88 are shown. DCE = dynamic contrast enhanced, T1w = T1 weighted, TRICKS = Time‐Resolved Imaging of Contrast KineticS. Figure S6. Gas T1 exponential decay of dissolved‐phase signal at inspired oxygen levels (FiO2) of 40% (fit in red) and 100% (fit in blue). Table S1. Regional descriptive statistics of two oxygen l [file NBM-38-e70063-s001.docx]

**Supplementary**

**
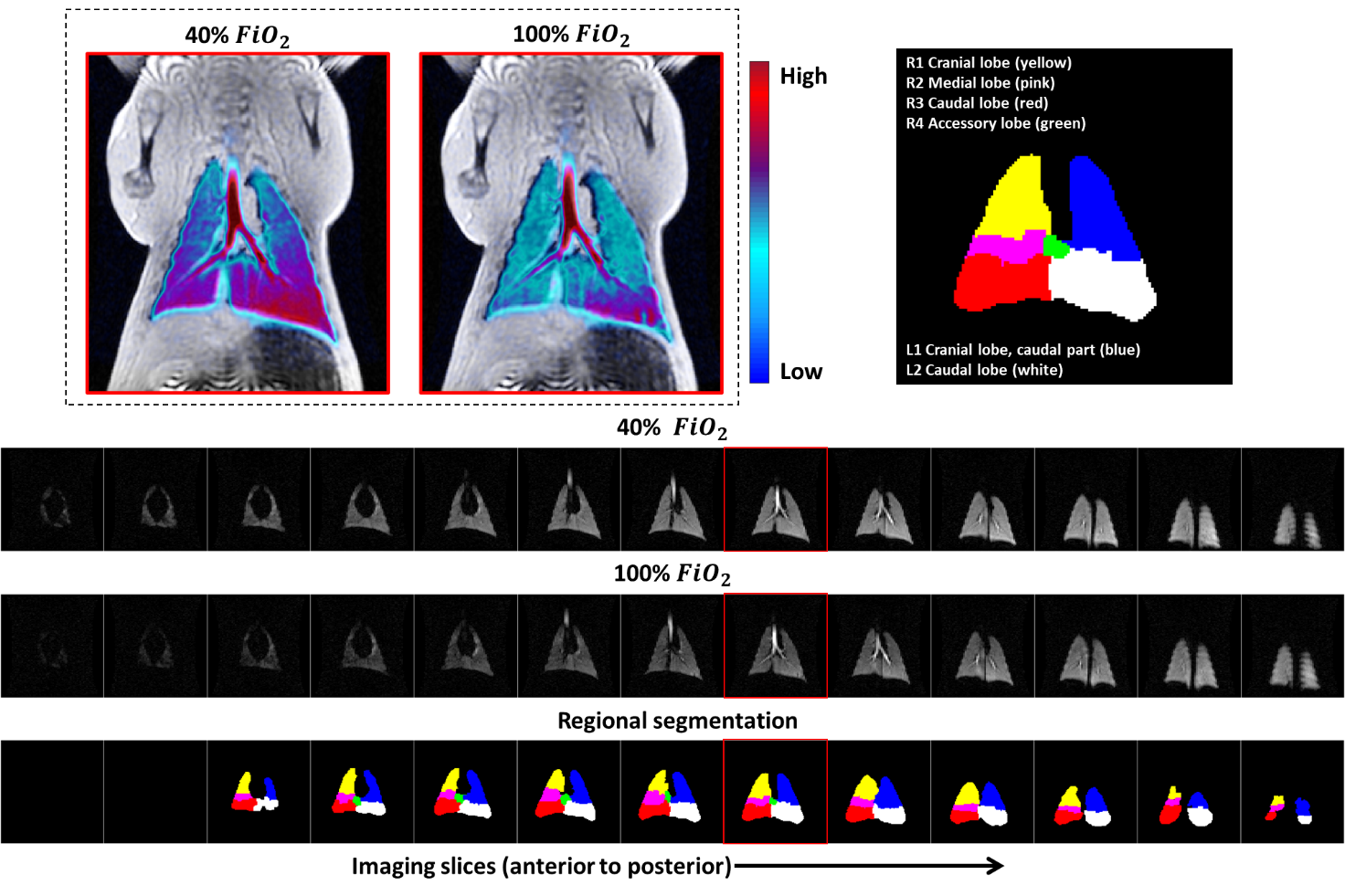
**

**Figure S1 –** Ventilation images at oxygen levels of 40% (top row) and 100% (middle row). T_1_-weigthed ^1^H MRI and ^129^Xe ventilation image overlay is shown in the top left which was used to calculate ventilation defect percentage. Regional segmentation mask of the animal lung lobes (R1-R4 and L1-L2) is shown in the top right. Corresponding image slices for segmentation, 40% and 100% oxygen concentrations are indicated with a red border.


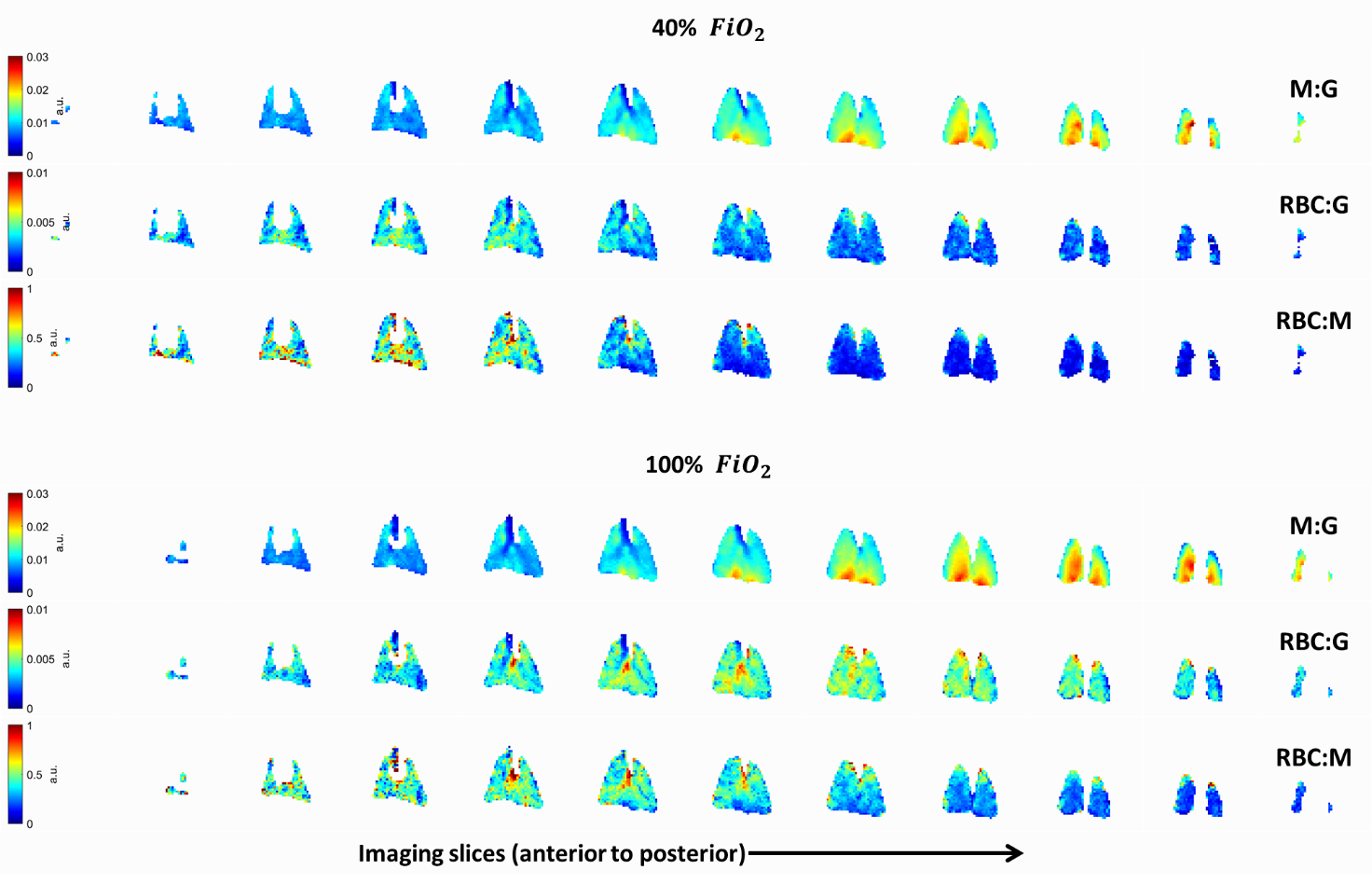


**Figure S2 –** Dissolved phase images of gas, membrane, and RBC with ratios acquired at fractional inspired oxygen levels (FiO_2_) of 40% (top six rows) and 100% (bottom six rows). M:G = membrane/gas, RBC:G = RBC/gas, RBC:M = RBC/membrane. Images have been masked based on the gas signal. G = gas, M = membrane, RBC = red blood cells.


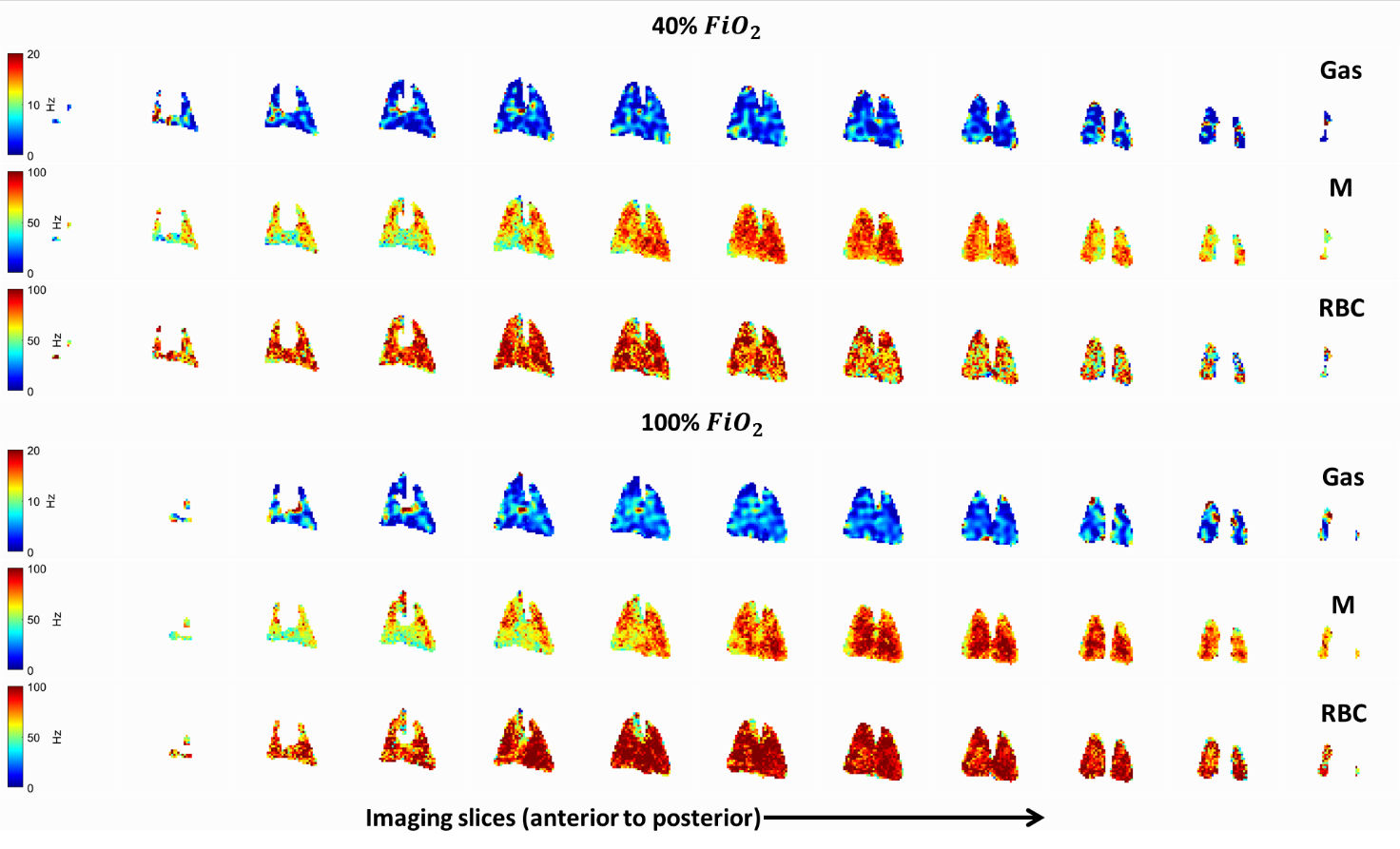


**Figure S3 –** Linewidth of gas, membrane and RBC peaks determined from the AMARES quantification at fractional inspired oxygen levels (FiO_2_) of 40% (top 3 rows) and 100% (bottom 3 rows). AMARES = Advanced Method for Accurate, Robust, and Efficient Spectral. M = membrane, RBC = red blood cells.


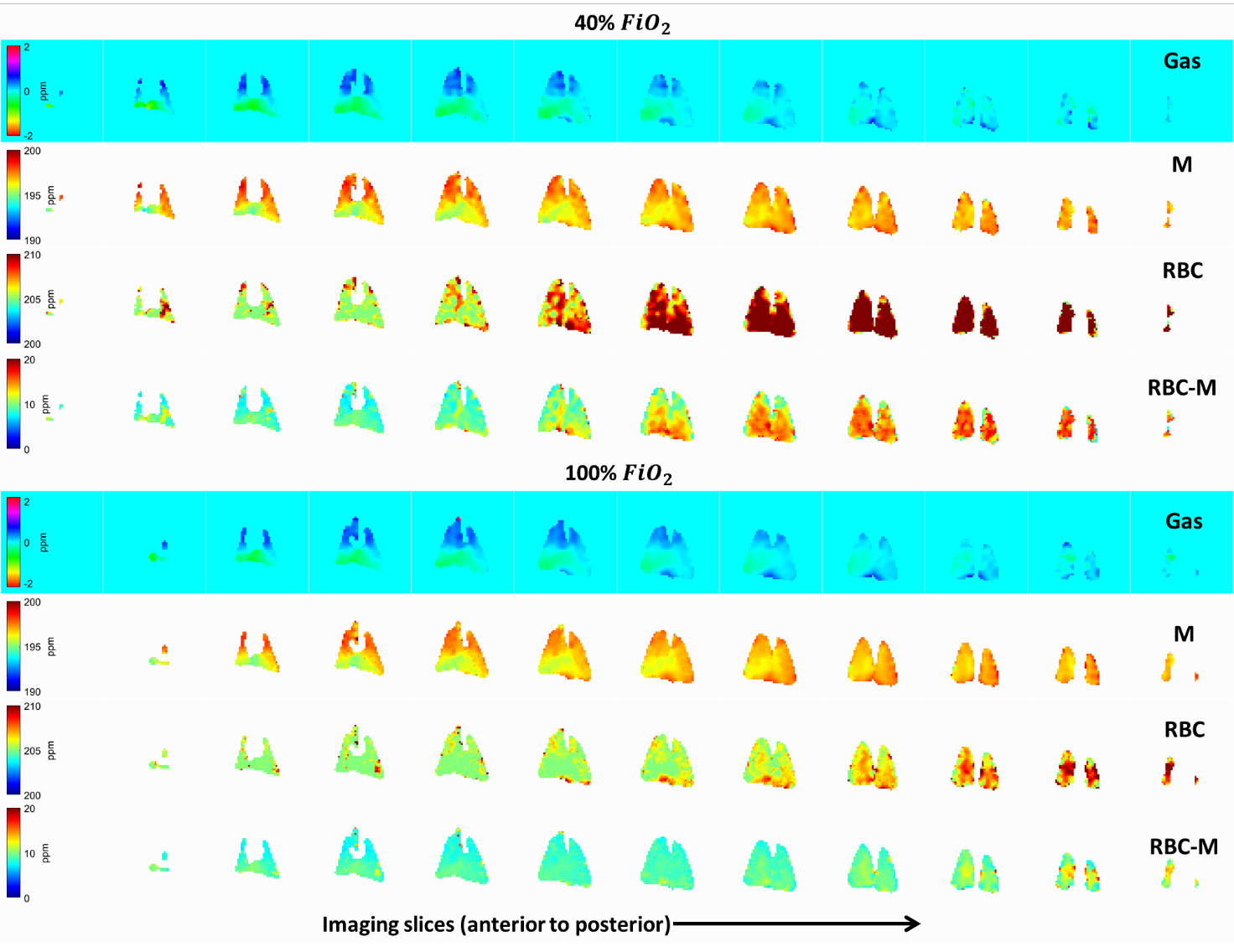


**Figure S4 –** Chemical shift images of gas, membrane, and RBC at fractional inspired oxygen levels (FiO_2_) 40% (top rows) and 100% (bottom rows). The chemical shift difference between RBC and membrane is shown at the last row in each oxygen section. M = membrane, RBC = red blood cells.


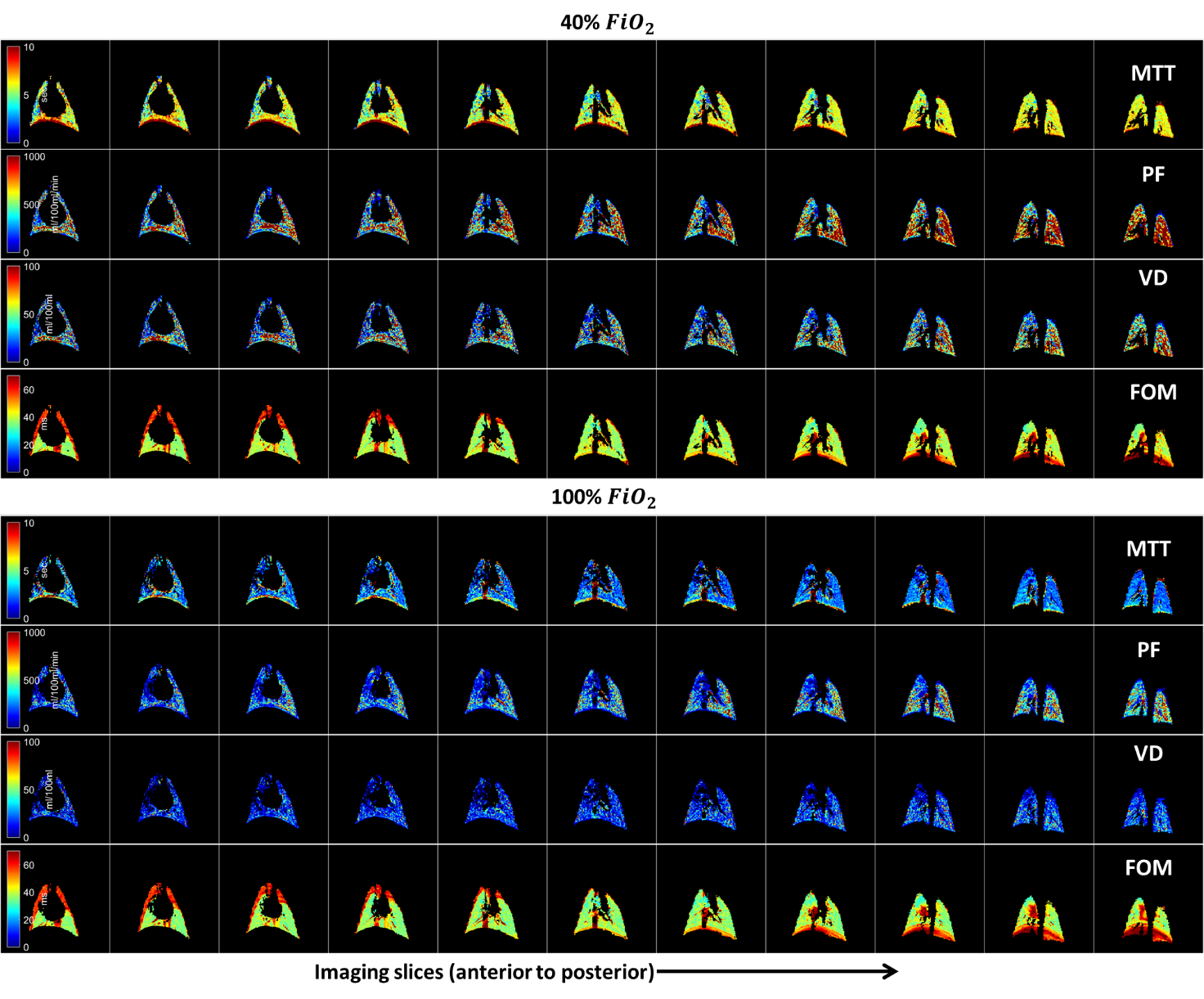


**Figure S5** **–** Perfusion images calculated from the TRICKS T_1_w DCE images masked to the lung. Calculated images include mean transit time (MTT), plasma flow (PF), volume of distribution (VD) and first order moment (FOM) acquired at fractional inspired oxygen levels (FiO_2_) of 40% (top four rows) and 100% (bottom four rows). The central 10 slices of 88 are shown. DCE = dynamic contrast enhanced, T_1_w = T_1_ weighted, TRICKS = Time-Resolved Imaging of Contrast KineticS.


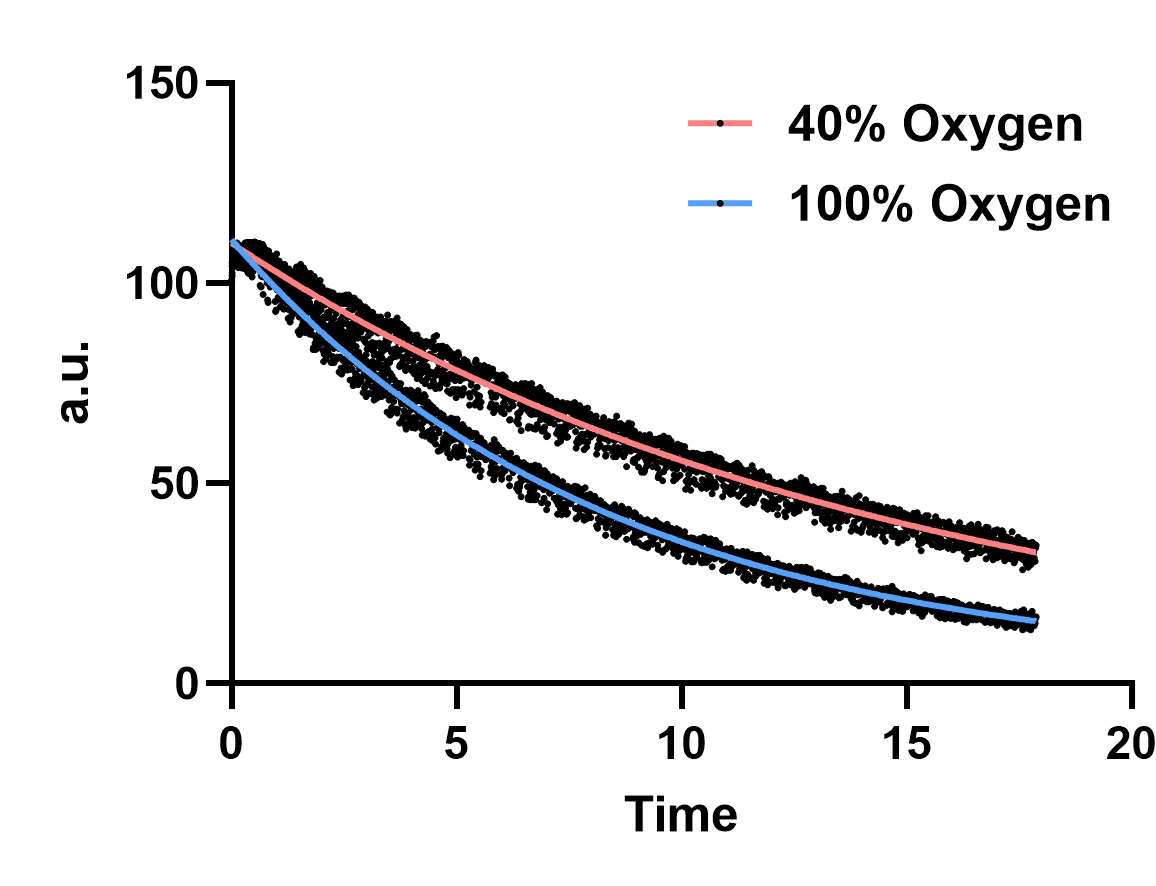


**Figure S6** **–** Gas T1 exponential decay of dissolved-phase signal at inspired oxygen levels (FiO_2_) of 40% (fit in red) and 100% (fit in blue).

***Table S1 – Regional descriptive statistics of two oxygen levels in dissolved phase ratios, chemical shifts, linewidth, and perfusion.***

|  | | **Lung lobe** | | | | | |
| --- | --- | --- | --- | --- | --- | --- | --- |
|  | **Unit** | **R1** | **R2** | **R3** | **R4** | **L1** | **L2** |
| **M:GAS** | a.u. |  |  |  |  |  |  |
| FiO_2_ 40% |  | 0.0094 ±  0.0009 | 0.0099 ±  0.0006 | 0.0127 ±  0.0005 | 0.0111 ±  0.0005 | 0.0130 ±  0.0004 | 0.0144 ±  0.0004 |
| FiO_2_ 100% |  | 0.0102 ±  0.0007 | 0.0107 ±  0.0003 | 0.0138 ±  0.0002 | 0.0121 ±  0.0004 | 0.0141 ±  0.0003 | 0.0158 ±  0.0006 |
| **RBC:GAS** | a.u. |  |  |  |  |  |  |
| FiO_2_ 40% |  | 0.0023 ±  0.0003 | 0.0024 ±  0.0003 | 0.0030 ±  0.0002 | 0.0027 ±  0.0002 | 0.0031 ±  0.0002 | 0.0034 ±  0.0002 |
| FiO_2_ 100% |  | 0.0031 ±  0.0004 | 0.0032 ±  0.0004 | 0.0041 ±  0.0003 | 0.0037 ±  0.0003 | 0.0043 ±  0.0003 | 0.0047 ±  0.0003 |
| **RBC:M** | a.u. |  |  |  |  |  |  |
| FiO_2_ 40% |  | 0.2409 ±  0.0215 | 0.2446 ±  0.0172 | 0.2864 ±  0.0065 | 0.2713 ±  0.0072 | 0.2929 ±  0.0117 | 0.3126 ±  0.0135 |
| FiO_2_ 100% |  | 0.2778 ±  0.0218 | 0.2848 ±  0.0162 | 0.3458 ±  0.0098 | 0.3238 ±  0.0097 | 0.3587 ±  0.0150 | 0.3866 ±  0.0171 |
| **Gas CS** | ppm |  |  |  |  |  |  |
| FiO_2_ 40% |  | 0.29 ±  0.10 | 0.21 ±  0.10 | 0.08 ±  0.10 | 0.08 ±  0.10 | 0.10 ±  0.10 | 0.06 ±  0.09 |
| FiO_2_ 100% |  | 0.35 ±  0.11 | 0.27 ±  0.11 | 0.15 ±  0.10 | 0.16 ±  0.10 | 0.17 ±  0.10 | 0.13 ±  0.09 |
| **M CS** | ppm |  |  |  |  |  |  |
| FiO_2_ 40% |  | 197.42 ±  0.16 | 197.23 ±  0.19 | 196.87 ±  0.19 | 196.83 ±  0.21 | 196.92 ±  0.20 | 196.83 ±  0.18 |
| FiO_2_ 100% |  | 197.40 ±  0.24 | 197.23 ±  0.23 | 196.92 ±  0.23 | 196.91 ±  0.24 | 196.99 ±  0.24 | 196.90 ±  0.20 |
| **RBC CS** | ppm |  |  |  |  |  |  |
| FiO_2_ 40% |  | 209.35 ±  0.45 | 209.23 ±  0.47 | 209.03 ±  0.35 | 208.93 ±  0.32 | 209.01 ±  0.36 | 208.93 ±  0.34 |
| FiO_2_ 100% |  | 206.83 ±  0.51 | 206.74 ±  0.45 | 206.51 ±  0.36 | 206.47 ±  0.38 | 206.48 ±  0.41 | 206.43 ±  0.36 |
| **Gas LW** | Hz |  |  |  |  |  |  |
| FiO_2_ 40% |  | 4.15 ±  1.84 | 4.33 ±  1.46 | 4.40 ±  0.91 | 4.48 ±  0.83 | 4.46 ±  0.74 | 4.31 ±  0.74 |
| FiO_2_ 100% |  | 4.89 ±  1.41 | 4.99 ±  1.29 | 4.92 ±  0.83 | 5.09 ±  0.73 | 5.02 ±  0.74 | 4.93 ±  0.66 |
| **M LW** | Hz |  |  |  |  |  |  |
| FiO_2_ 40% |  | 73.82 ±  3.38 | 74.59 ±  3.52 | 72.92 ±  3.15 | 72.36 ±  3.29 | 74.10 ±  3.29 | 74.19 ±  3.03 |
| FiO_2_ 100% |  | 77.71 ±  3.28 | 77.82 ±  3.23 | 75.64 ±  3.29 | 74.97 ±  3.20 | 76.36 ±  3.18 | 76.49 ±  3.01 |
| **RBC LW** | Hz |  |  |  |  |  |  |
| FiO_2_ 40% |  | 74.18 ±  4.51 | 75.22 ±  3.33 | 76.15 ±  2.57 | 76.12 ±  2.73 | 76.53 ±  2.65 | 77.06 ±  2.32 |
| FiO_2_ 100% |  | 77.96 ±  3.80 | 80.08 ±  3.18 | 82.31 ±  3.39 | 81.72 ±  3.35 | 82.61 ±  3.31 | 84.02 ±  2.93 |
| **MTT** | s |  |  |  |  |  |  |
| FiO_2_ 40% |  | 11.87 ±  11.72 | 10.22 ±  8.22 | 8.86 ±  5.30 | 10.06 ±  7.16 | 9.38 ±  6.27 | 8.31 ±  4.39 |
| FiO_2_ 100% |  | 4.63 ±  0.49 | 5.02 ±  1.39 | 5.37 ±  1.52 | 5.46 ±  1.56 | 5.10 ±  1.38 | 5.19 ±  1.48 |
| **PF** | ml/100ml/min |  |  |  |  |  |  |
| FiO_2_ 40% |  | 557 ±  178 | 571 ±  169 | 571 ±  148 | 569 ±  147 | 597 ±  163 | 613 ±  179 |
| FiO_2_ 100% |  | 394 ±  121 | 406 ±  107 | 409 ±  71 | 404 ±  65 | 442 ±  81 | 451 ±  70 |
| **VD** | ml/100ml |  |  |  |  |  |  |
| FiO_2_ 40% |  | 47.61 ±  12.58 | 50.90 ±  16.08 | 53.63 ±  16.52 | 54.32 ±  16.41 | 53.78 ±  15.83 | 55.04 ±  16.19 |
| FiO_2_ 100% |  | 26.13 ±  9.05 | 27.68 ±  10.51 | 29.97 ±  10.72 | 30.43 ±  11.13 | 31.10 ±  12.01 | 31.62 ±  12.18 |
| **FOM** | ms |  |  |  |  |  |  |
| FiO_2_ 40% |  | 48.86 ±  4.17 | 48.56 ±  4.56 | 48.81 ±  4.67 | 49.07 ±  4.62 | 49.06 ±  5.01 | 49.94 ±  4.99 |
| FiO_2_ 100% |  | 49.48 ±  5.45 | 49.69 ±  5.99 | 50.01 ±  5.88 | 50.38 ±  5.70 | 50.17 ±  5.97 | 50.88 ±  6.09 |

Fractional inspired oxygen level (FiO_2_), Membrane (M), Red blood cells (RBC), Chemical shift (CS), Linewidth (LW), Mean transit time (MTT), plasma flow (PF), volume of distribution (VD) and first order moment (FOM). R1 – right cranial lobe, R2 – right middle lobe, R3 – right caudal lobe, R4 – right accessory lobe, L1 – left cranial lobe, L2 – left caudal lobe.
